# Decoding the Cardiac Immune Microenvironment and Fibroblast Crosstalk in Radiotherapy Combined with Immunotherapy‐Induced Cardiac Fibrosis Based on Single‐Cell Transcriptomic Analysis

**DOI:** 10.1002/advs.202519216

**Published:** 2026-02-20

**Authors:** Yuxi Luo, Ying Yu, Zhimin Zeng, Yanxin Chen, Yali Yi, Zhiqin Lu, Fujuan Zeng, Peng Xu, Daya Luo, Leifeng Chen, Anwen Liu

**Affiliations:** ^1^ Department of Oncology, The Second Affiliated Hospital, Jiangxi Medical College Nanchang University Nanchang Jiangxi Province China; ^2^ Jiangxi Province Key Laboratory of Precision Cell Therapy, The Second Affiliated Hospital, Jiangxi Medical College Nanchang University Nanchang Jiangxi Province China; ^3^ Radiation Induced Heart Damage Institute, Jiangxi Medical College Nanchang University Nanchang Jiangxi Province China; ^4^ Department of Biochemistry and Molecular Biology, School of Basic Medical Sciences Nanchang University Nanchang Jiangxi Province China

**Keywords:** cardiac fibrosis, fibroblasts, immune checkpoint inhibitor, immune microenvironment, thoracic radiotherapy

## Abstract

**Purpose**: The combination of radiotherapy and immunotherapy (radioimmunotherapy) shows promising antitumor efficacy but raises cardiotoxicity concerns. The underlying mechanisms remain unclear.

**Methods**: Preclinical models were used to assess cardiac function at day 28, 3 months, and 5 months post radioimmunotherapy intervention. The scRNA‐seq and molecular experiments were conducted. IL‐6 knockout mice and tocilizumab (IL‐6R inhibitor) were used for targeted interventions.

**Results**: Radioimmunotherapy exacerbated cardiac fibrosis and enhanced fibroblast‐immune cell crosstalk, accompanied by robust activation of IL‐6 signaling predominantly derived from fibroblasts. Elevated serum IL‐6 levels were also observed in patients receiving combined thoracic radiotherapy and immunotherapy. Both IL‐6 knockout and tocilizumab treatment effectively alleviated acute cardiac injury, inflammation, and fibrosis. Notably, tocilizumab likely inhibits the IL‐6^+^ fibroblasts‐mediated activation of themselves and CCR2^+^ macrophages, which these subsets exhibit enhanced pro‐fibrotic scores in radioimmunotherapy‐induced cardiac damage. Moreover, alterations in immune checkpoint molecules were observed in the cardiac microenvironment following radioimmunotherapy. Macrophages with high IL‐6 signaling activity exhibited elevated CD86 expression, which was reduced upon tocilizumab treatment.

**Conclusions**: Our study identifies fibroblast‐immune cell interactions, particularly IL‐6‐mediated fibroblast‐macrophage crosstalk, as a key mechanism in radioimmunotherapy‐induced cardiac fibrosis. Tocilizumab, an IL‐6R inhibitor, demonstrates therapeutic potential to attenuate this cardiotoxicity.

## Graphical abstract

This study highlights that radioimmunotherapy drives crosstalk between fibroblasts and immune cells (especially macrophages) in the cardiac microenvironment, with IL‐6 as the key mediator, and tocilizumab alleviates cardiac fibrosis by targeting this interplay.

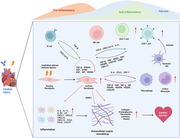

## 1 Introduction

In recent times, a profound comprehension of the tumor immune microenvironment and immune evasion mechanisms has sparked a paradigm shift in cancer treatment, with immune checkpoint inhibitor (ICI) regimen targeting PD‐1/PD‐L1 and CTLA‐4 has revolutionized the treatment paradigm for malignant tumors [1]. Clinical trials and real‐world applications have showcased the promise of combined therapy strategy involving ICI, notably immunotherapy combined with radiotherapy. The strategy, exemplified by the PACIFIC trial and ongoing research endeavors, has highlighted that administering anti‐PD‐1/PD‐L1 therapy subsequent to chemoradiotherapy notably enhances overall survival of lung cancer patients [2, 3, 4]. However, ICI therapy‐mediated immune toxicity, coupled with the adverse reactions induced by radiotherapy, may lead to cumulative damage to vital thoracic organs, particularly the lungs and heart, ultimately limiting the efficacy of the combined approach and detracting from the life quality for cancer patients [5].

Radiation therapy is well‐documented to cause tissue damage in the irradiated area, and heart damage may be unavoidable during thoracic radiotherapy. As the most severe long‐term complications, radiation‐induced heart damage (RIHD) encompasses coronary atherosclerosis, myocardial injury, myocardial fibrosis, pericardial diseases, conduction system disorders, and heart valve injuries, all of which significantly compromise patients' long‐term survival prospects [6]. Immune‐related adverse events (irAEs) triggered by ICI involve multiple organ damage, specifically in the heart, leading to cardiovascular events such as myocarditis, pericarditis, vasculitis, coronary artery spasm, and arrhythmias [7]. Notably, ICI‐induced myocarditis garners significant attention due to its extremely high mortality rate, ranging from approximately 35% to 67% [7, 8]. Cardiac fibrosis emerges as a shared pathological process underlying cardiac injury induced by immunotherapy or radiotherapy. While it initially serves a protective role, unresolved fibrosis can lead to ventricular stiffness, heart failure, and sudden death due to arrhythmia [9].

While some studies have delved into the mechanisms underlying cardiac injury associated with radiotherapy or ICI alone, research on the risk and potential mechanisms of cardiac damage resulting from their combined treatment modality is still in its infancy. Notably, two clinical studies focusing on locally advanced non‐small cell lung cancer (NSCLC), PACIFIC and Gemstone‐301, revealed a higher incidence of cardiovascular events in patients receiving ICI maintenance therapy after radical chemoradiotherapy compared to those in the placebo maintenance group [5]. Furthermore, the Keynote‐799 clinical trial demonstrated promising antitumor activity with the combination of radiotherapy and immunotherapy, yet also reported two cases of myocarditis [10]. Regarding the mechanisms underlying cardiotoxicity induced by the combination of radiotherapy and ICI, limited researches suggests that it may involve increased infiltration of CD8^+^ T lymphocytes within the myocardial microenvironment, leading to an enhanced autoimmune response and subsequently exacerbating cardiac damage [11, 12]. Given the growing prevalence of the combined radiotherapy and ICI treatment modality in oncology, a thorough investigation into the intrinsic mechanisms underlying its cardiac toxicity is crucial for preventing and managing cardiovascular injuries associated with this combination therapy.

To comprehensively decipher the complex interplay underlying cardiac injuries arising from such combined treatment, we construct single‐cell transcriptomics sequencing (scRNA‐seq) atlas based on radioimmunotherapy‐induced mouse model, derived a comprehensive view of cardiac constituents and their subsets proportion, and unravel the intricate signaling cascades and regulatory networks between fibroblasts and immune cells within the cardiac microenvironment. Furthermore, we strive to identify potential therapeutic targets to mitigate cardiac fibrosis by leveraging clinical and preclinical evidence.

## 2 Results

### 2.1 Radioimmunotherapy Triggers a Time‐Dependent Transition toward Irreversible Cardiac Dysfunction

**FIGURE 1:**
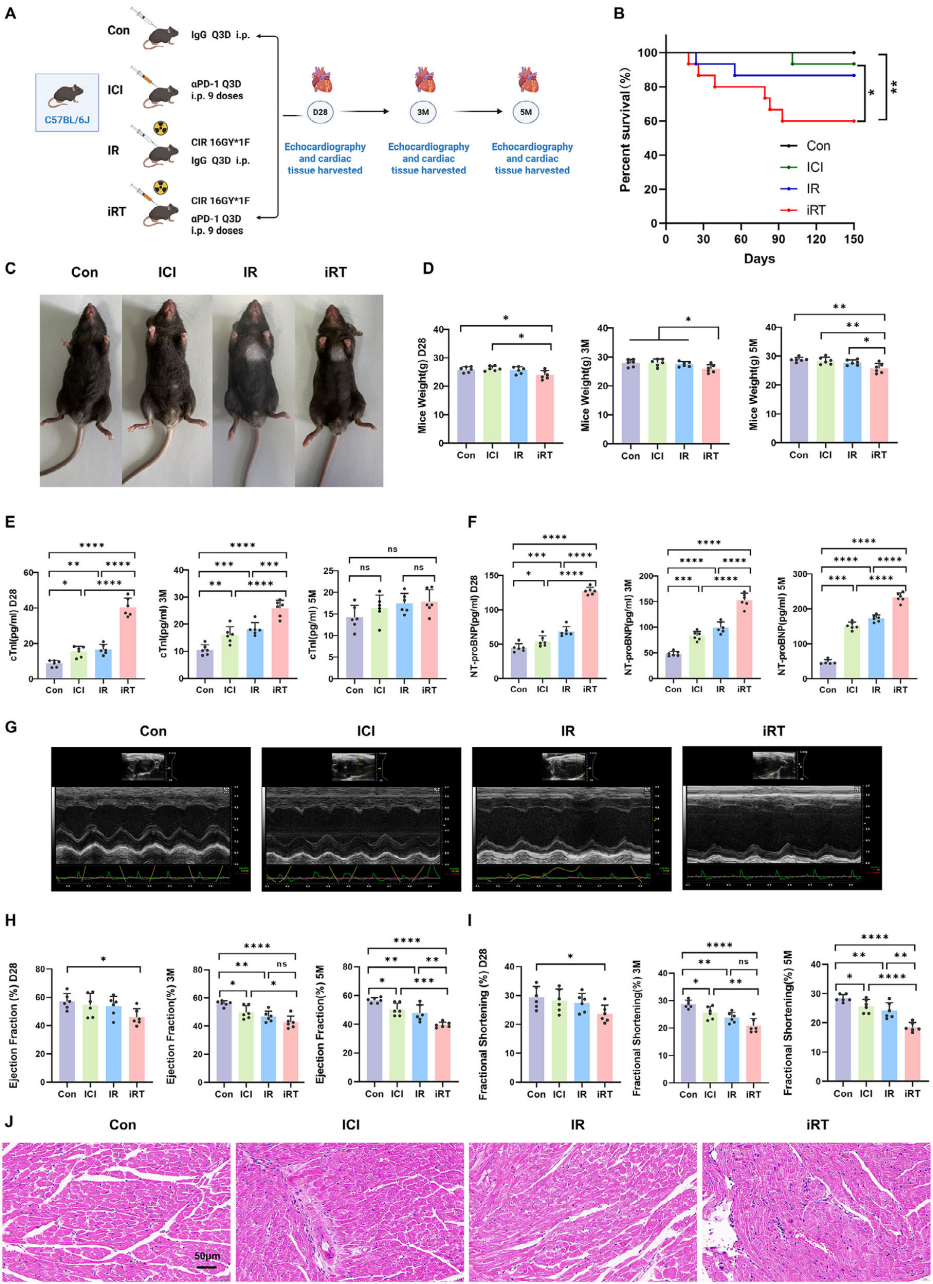
Temporal progression of radioimmunotherapy‐induced cardiac injury from acute damage to chronic remodeling. (A) Flowchart of the mouse model construction for cardiac injury induced by the CIR and ICI (*n* = 18 per group; *n* = 6 per time point). (B) Survival curves of mice across from Con, ICI, IR, and iRT groups over 150 days (*n* = 15 per group). (C) Irradiation‐induced skin reactions across four groups at day 28 post‐intervention. (D) Changes in body weight of mice in Con, ICI, IR, and iRT groups at day 28, 3 months, and 5 months post‐intervention (*n* = 6 per group). (E) Serum cardiac troponin I (cTnI) levels of mice from Con, ICI, IR, and iRT groups at day 28, 3 months, and 5 months post‐intervention detected by ELISA (*n* = 6 per group). (F) Serum N‐terminal pro‐B‐type natriuretic peptide (NT‐proBNP) levels of mice from Con, ICI, IR, and iRT groups at day 28, 3 months, and 5 months post‐intervention detected by ELISA (*n* = 6 per group). (G) Representative M‐mode echocardiographic images of mice from Con, ICI, IR, and iRT groups at 5 months post‐intervention. (H) Left ventricular ejection fraction (LVEF) measured by echocardiography from Con, ICI, IR, and iRT groups at day 28, 3 months, and 5 months post‐intervention (*n* = 6 per group). (I) Left ventricular fractional shortening (LVFS) measured by echocardiography from Con, ICI, IR, and iRT groups at day 28, 3 months, and 5 months post‐intervention (*n* = 6 per group). (J) Hematoxylin and eosin (H&E) staining of cardiac tissues from mice in Con, ICI, IR, and iRT groups at day 28 post‐intervention, scale bar = 50 µm. Data are presented as mean ± standard deviation (SD). The one‐way analysis of variance (ANOVA) was performed to compare data. ns: not significant, *p < 0.05, **p < 0.01, ***p < 0.001, ****p < 0.0001.

Building upon the insights from our prior research on RIHD and immune therapy‐related heart damage [5], we established the murine model of radioimmunotherapy‐induced cardiac injury, including the cardiac irradiation group (IR), PD‐1 inhibitor treatment group (ICI), combined irradiation and PD‐1 inhibitor treatment group (iRT), and untreated control group (Con) (Figure 1A). To investigated the impact of cardiac irradiation combined with PD‐1 inhibitor on cardiac tissues and its underlying mechanisms in vivo, we systematically evaluated the dynamic changes in cardiac injury across the four groups of mice at day 28, 3 months, and 5 months after intervention, by utilizing echocardiography, serum biomarkers of cardiac troponin I (cTnI) and N‐terminal pro‐B‐type natriuretic peptide (NT‐proBNP), hematoxylin and eosin (H&E), and Masson's trichrome staining (Figure 1A).

Our findings revealed the iRT group exhibited significantly higher acute and long‐term mortality compared with the ICI, IR, and Con groups (Figure 1B). At day 28 post‐iRT intervention, mice in the IR and iRT groups displayed marked radiation‐induced skin reactions over the chest wall at the irradiation site, indicative of direct local tissue damage (Figure 1C). At day 28 post‐intervention, mice in the iRT group exhibited a significant decrease in body weight compared to the ICI and Con groups. Subsequently, at 3 and 5 months, mice in the iRT group continued to demonstrate marked reduction in body weight relative to the IR, ICI, and Con groups, suggesting detrimental long‐term effects of combined radioimmunotherapy on overall health status (Figure 1D).

Serum analysis revealed significantly elevated cTnI and NT‐proBNP levels in the iRT group compared with the ICI, IR, and Con groups at day 28, while both the ICI and IR groups also showed higher levels than Con group (Figure 1E,F). At the 3‐month time point, both cTnI and NT‐proBNP levels remained significantly elevated in the iRT group compared to all other groups. However, while cTnI levels decreased relative to day 28 and showed diminished intergroup variability, NT‐proBNP levels continued to rise, resulting in more pronounced divergence between the groups (Figure 1E,F). By 5 months, cTnI levels no longer differed significantly among groups, whereas NT‐proBNP exhibited the most pronounced group differences across the entire time course (Figure 1E,F). In addition, echocardiographic assessment revealed substantial declines in left ventricular function in the iRT group, marked by reduced left ventricular ejection fraction (LVEF), and fractional shortening (LVFS) compared to the Con group at day 28 post‐intervention (Figure 1H,I). At both 3 and 5 months, echocardiographic evaluation demonstrated further declines in LVEF and LVFS in the iRT group relative to the Con group, with the differences becoming more pronounced over time (Figure 1G–I). In addition, although the impairment was most evident in the iRT group, mice receiving either IR or ICI alone also exhibited significant reductions in LVEF and LVFS compared with Con group (Figure 1H,I). Corroborating these findings, H&E staining at the acute phase (day 28) revealed that myocardial tissues in the intervention groups exhibited significant structural disorganization compared to the control group. Cardiac tissues in the experimental group exhibited prominent vacuolar degeneration and swelling of myocardial cells compared to the control group; additionally, increased inflammatory cell infiltration was observed in the myocardial interstitium of the iRT and ICI groups (Figure 1J).

These findings indicate that combined radioimmunotherapy may accelerate acute cardiac injury through synergistic effects, which subsequently progresses into a chronic injury phase characterized by structural remodeling and reparative processes. This dynamic progression not only underscores the potential long‐term detrimental effects of combined radiotherapy and ICI on cardiac tissue, but also emphasizes the importance of early intervention and long‐term cardiac monitoring in mitigating chronic toxicity. Furthermore, single‐modality interventions with either IR or ICI alone are sufficient to elicit sustained cardiac functional decline.

### 2.2 Aggravation of Myocardial Fibrosis in Mice through CIR Combined with ICI

**FIGURE 2:**
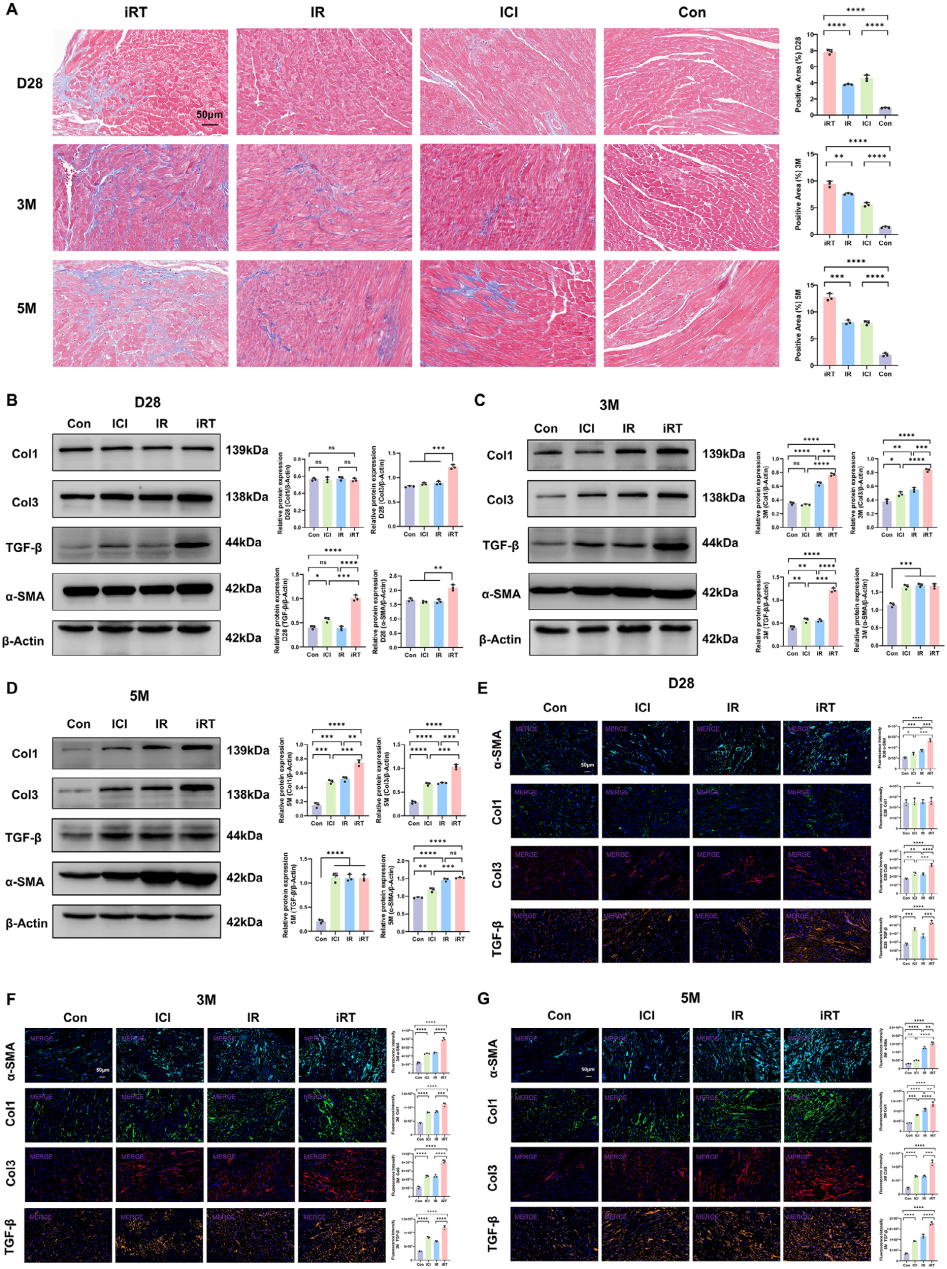
Combined radioimmunotherapy promotes time‐dependent progression of cardiac fibrosis. (A) Masson's trichrome staining of cardiac tissues from Con, ICI, IR, and iRT groups at day 28, 3 months, and 5 months post‐intervention, scale bar = 50 µm; quantitative analysis of collagen deposition at each time point. (B–D) Western blot analysis of Col1, Col3, α‐SMA, and TGF‐β protein levels in myocardial tissues from Con, ICI, IR, and iRT groups at day 28 (B), 3 months (C), 5 months (D) post‐intervention; quantitative analysis of protein expression levels. (E–G) Immunofluorescence staining of α‐SMA, TGF‐β, Col1, and Col3 in cardiac tissues of Con, ICI, IR, and iRT groups at day 28 (E), 3 months (F), 5 months (G) post‐intervention, scale bar = 50 µm; quantitative histograms of corresponding markers. Data are presented as mean ± SD. The one‐way ANOVA was performed to compare data. ns: not significant, *p < 0.05, **p < 0.01, ***p < 0.001, ****p < 0.0001, *n* = 3 per group.

Cardiac fibrosis, as a critical pathophysiological manifestation of radiation‐ and ICI‐induced cardiac injury, prompted us to further evaluate the specific impact of combined radioimmunotherapy on myocardial fibrosis. We first assessed the extent of collagen deposition in cardiac tissues across the four groups using Masson's trichrome staining. The results demonstrated that mice in the iRT group exhibited more pronounced collagen fiber deposition compared to those in the Con, ICI, and IR groups (Figure 2A). Furthermore, from day 28 through the 3‐ and 5‐month time points, the extent of collagen deposition increased progressively over time in the iRT, IR, and ICI groups (Figure 2A). Given the cardioprotective effects of estrogen in females, we extended our investigation to female mouse models subjected to combined radioimmunotherapy (Figure S1A). Notably, we observed that the combined intervention also induced acute cardiac injury and fibrotic responses during the early phase (day 28) following intervention. However, no acute mortality events were observed in female mice within 28 days following radioimmunotherapy, and body weights remained stable without significant differences among groups (Figure S1B–H). The apparent attenuation of acute mortality and the preservation of body weight in female mice after combined radioimmunotherapy may reflect the cardioprotective effects of ovarian estrogens [13]. However, the specific effects and mechanisms remain to be further verified in future clinical and basic research.

To further quantitatively assess the degree of myocardial fibrosis, we employed Western blot and immunofluorescence staining analysis. At day 28 post‐intervention, both assays indicated significantly elevated levels of alpha‐smooth muscle actin (α‐SMA), transforming growth factor‐β (TGF‐β), and Collagen Type III (Col3) in cardiac tissues of the iRT group compared to the Con group. In contrast, no significant differences were observed in Collagen Type I (Col1) expression among the groups (Figure 2B). Immunofluorescence results also showed modest increases in α‐SMA, TGF‐β, and Col3 levels in the ICI and IR groups relative to controls, though to a lesser extent than in the iRT group (Figure 2E). As the experiment progressed to 3‐ and 5‐ month time points, Western blot and immunofluorescence analyses revealed a marked induction of α‐SMA, TGF‐β, Col1, and Col3 expression in the iRT group compared to the IR, ICI, and Con groups (Figure 2C,D,F,G). In summary, our study underscores that concurrent whole‐heart irradiation and PD‐1 inhibition in mice triggers acute cardiac dysfunction and tissue disruption, progressing to pronounced, time‐dependent fibrotic lesions over day 28 to 5 months, underscoring the need for early intervention and sustained monitoring of cardiotoxicity in the setting of radioimmunotherapy. Nevertheless, the underlying mechanisms of the cardiac fibrosis, stemming from the combined administration of radiotherapy and immunotherapy, remain largely unexplored.

### 2.3 Single‐Cell Transcriptomics Atlas of Cardiac Fibrosis in Mice Induced by CIR Combined with PD‐1 Inhibitor

**FIGURE 3:**
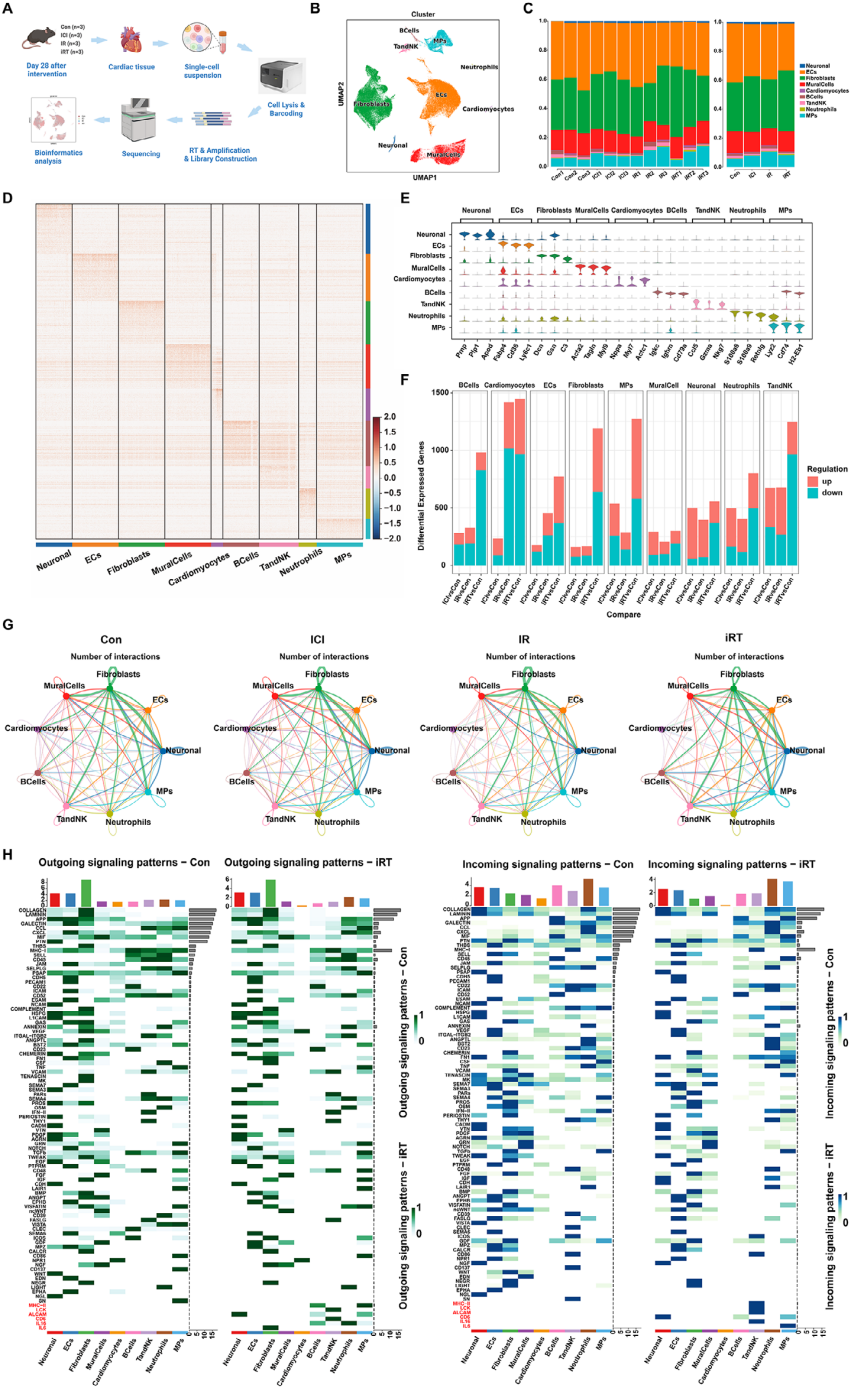
The single‐cell transcriptomics landscape of mouse cardiac fibrosis treated with ICI and/or CIR. (A) Schematic workflow and grouping information for single‐cell transcriptomics sequencing (scRNA‐seq) using the 10× Illumina novaseq 6000 platform (*n* = 3 per group). (B) Integrated Uniform Manifold Approximation and Projection (UMAP) visualization and cell type annotation of single‐cell transcriptomes analysis from 11 1566 mouse cardiac cells treated with ICI or/and CIR. (C) The proportion of nine cell types in the hearts of mice across 12 mouse heart samples from Con, ICI, IR, and iRT groups (left), and after merging by intervention group (right). (D) Heatmap of the top 500 differentially expressed genes (DEGs) in each cluster identified through unsupervised clustering of all the cardiac cells. Blue indicates lower expression, and red indicates higher expression. The expression scale is shown on the right. (E) Stacked violin plot of the top 3 marker genes for each cell type in scRNA‐seq of cardiac fibrosis induced by the intervention of ICI and/or CIR. (F) The number of DEGs in each cell type of the mouse hearts for ICl vs Con, IR vs Con, and iRT vs Con. Red blocks represent upregulated genes, while teal blocks represent downregulated genes. (G) The network diagram of cell–cell interaction number in myocardial tissues of the Con, ICl, IR, and iRT groups at day 28 post‐intervention. The number of ligand‐receptor interaction pairs is indicated according to line width. (H) The heatmap showing possible efferent (left) and afferent (right) signaling pathways between nine cell types in the Con and iRT groups at day 28 of intervention.

To systematically elucidate the cellular and molecular mechanisms underlying cardiac fibrosis induced by the combined radioimmunotherapy, we performed scRNA‐seq analysis on mouse myocardial tissue obtained on day 28 from four groups (Con, ICI, IR, and iRT groups), with three biological replicates per group and a total of 12 samples (Figure 3A). After stringent quality control, excluding red blood cells and filtering cells with > 20% mitochondrial genes or < 200 detected genes, we retained 111,566 high‐quality single cells for downstream analysis. Using the Scanpy v1.8.2 pipeline, we performed quality filtering, normalization, dimensionality reduction, and unsupervised clustering. Cell clusters were visualized via Uniform Manifold Approximation and Projection (UMAP). Based on classical marker genes (Table S1), we identified nine major cell types, including neuronal cells, endothelial cells (ECs), fibroblasts, mural cells, cardiomyocytes, B cells (BCells), T and NK cells (TandNK), neutrophils, and mononuclear phagocytes (MPs) (Figure 3B). Due to the large cell diameter and consequent low capture efficiency of scRNA‐seq, cardiomyocytes constituted only 1.73% of all cells. Stromal cells, including fibroblasts (38.15%), endothelial cells (35.77%), mural cells (13.88%), MPs (8.08%), and TandNK cells (1.14%), comprised the majority of the population (Figure 3C). Quantitative analysis of cellular composition revealed a marked increase in the proportion of fibroblasts in the ICI and iRT groups (Con: 33.48%; ICI: 38.28%; IR: 33.50%; iRT: 41.84%). Similarly, the proportion of MPs was elevated across all intervention groups (Con: 5.65%; ICI: 7.74%; IR: 10.64%; iRT: 8.38%), suggesting that both fibroblasts and MPs may play central roles in mediating fibrotic remodeling following radioimmunotherapy‐induced cardiac injury (Figure 3C).

We next compared transcriptional alterations across the nine major cardiac cell types among Con, ICI, IR, and iRT groups. Visualization of the top 500 differentially expressed genes (DEGs) via heatmap and the top three DEGs per cluster via violin plots revealed distinct transcriptional heterogeneities among cell types (Figure 3D,E). Further analysis of DEGs between each intervention group (ICI, IR, iRT) and Con within these nine cell types indicated that CIR or/and ICI intervention induced substantial transcriptomic alterations in fibroblasts, cardiomyocytes, ECs, MPs, TandNK cells, and B cells, with the most pronounced changes observed in the iRT versus Con groups (Figure 3F; Figure S2A–F and Table S2).

To explore intercellular communication, we performed a CellChat analysis of the global cellular network across four intervention conditions. Results indicated that fibroblasts exhibited the highest number of cellular interactions under all four intervention regimens, underscoring their central role in cardiac injury and fibrotic remodeling (Figure 3G). Further dissection of outgoing and incoming signals among the nine cell clusters in iRT versus Con groups identified fibroblasts as the primary signal senders, with macrophages and neutrophils serving as major recipients (Figure 3H). Beyond these global patterns, several treatment‐specific signaling pathways were selectively activated in the iRT group versus Con group, including MHC‐II_CD4, LCK_CD8A_CD8B1, ALCAM_CD6, IL16_CD4, and IL‐6_IL‐6R. Specifically, the ligand IL16 exhibits specific enrichment in B cells, MPs, and neutrophils, while its receptor CD4 is exclusively expressed in TandNK cells. Similarly, MHC‐II shows selective accumulation in B cells and MPs, with their interacting receptor CD4 localized to TandNK cells. The ALCAM/CD6 axis demonstrated bidirectional specificity, with ALCAM expressed in neuronal cells, B cells and MPs, and CD6 limited to TandNK populations. LCK and the CD8A–CD8B1 receptor complex were co‐enriched within TandNK subsets, suggesting autocrine or paracrine signaling within these lymphocytes. Most importantly, IL‐6/IL‐6R signaling was predominantly characterized by a fibroblast‐to‐MP communication axis in the iRT group, wherein fibroblasts specifically produced IL‐6 and MPs exclusively expressed the IL‐6R receptor (Figure 3H). However, the specific regulatory effects and signaling pathways through which the intercellular crosstalk remodels the cardiac microenvironment in radioimmunotherapy‐induced fibrosis warrant further investigation.

### 2.4 Cardiac Fibroblasts Exhibit Significant Pro‐Fibrotic and Pro‐Inflammatory Properties in Mouse Hearts Induced by CIR Combined with ICI

**FIGURE 4:**
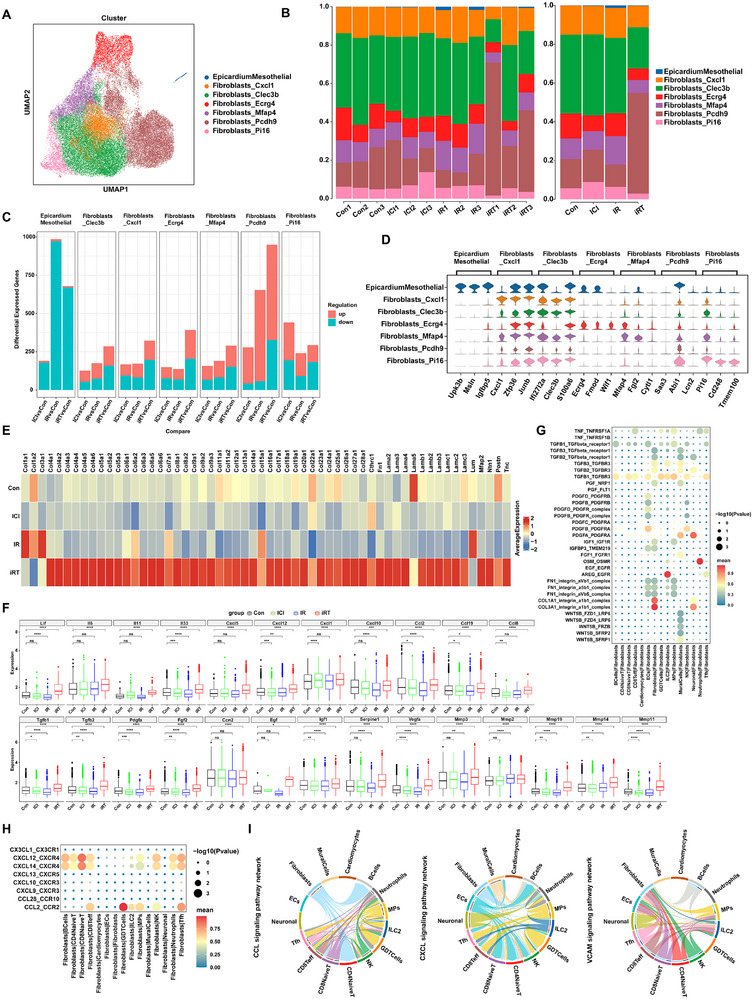
Cardiac fibroblasts exhibit significant pro‐fibrotic and pro‐inflammatory properties in mouse hearts subjected to ICI combined with CIR. (A) UMAP visualization of scRNA‐seq from fibroblasts in mouse hearts treated with ICI or/and CIR. (B) The proportion of fibroblast subclusters in mice hearts across 12 mouse heart samples from Con, ICI, IR, and iRT groups (left), and after merging by intervention group (right). (C) The number of DEGs in fibroblast subtypes of the mouse hearts for ICl vs Con, IR vs Con, and iRT vs Con. Red blocks represent upregulated genes, while teal blocks represent downregulated genes. (D) Stacked violin plot of the top 3 marker genes for fibroblast subtypes. (E) Heatmap of expression levels of extracellular matrix (ECM) molecules in fibroblasts from Con, ICI, IR, and iRT groups. (F) The box plots showing differential expression levels of pro‐fibrotic and fibrinolysis‐related factors in fibroblasts from cardiac tissues of Con, ICI, IR, and iRT groups. (G) The pro‐fibrotic interaction diagram of fibroblasts acting as receptor cells with other cell types in the iRT group. (H) The chemokine interaction diagram of fibroblasts acting as ligand cells with other cell types in the iRT group. (I) The chord diagram of the cell‐cell interactions networks of CCL, CXCL, and VCAM signaling pathway network in the iRT group. Data are presented as box plots showing the median, interquartile range, and potential outliers. Differences between groups were assessed using the nonparametric Wilcoxon rank‐sum test (F). ns: not significant, *p < 0.05, **p < 0.01, ***p < 0.001, ****p < 0.0001.

To elucidate the functional heterogeneity of fibroblasts in combined radioimmunotherapy‐induced cardiac fibrosis, we performed subcluster analysis on 42,246 fibroblasts obtained from scRNA‐seq data. Unsupervised clustering and annotation using Scanpy v1.8.2 identified seven distinct fibroblast subpopulations: Cxcl1^+^, Clec3b^+^, Ecrg4^+^, Mfap4^+^, Pcdh9^+^, Pi16^+^, and EpicardiumMesothelial^+^ fibroblasts (Figure 4A). Notably, the Pcdh9^+^ subpopulation was significantly expanded in the iRT group (Figure 4B). To visualize their transcriptional diversity, the heatmap of the top 100 genes and the violin plot of the top three genes were generated in each subcluster, which highlighted distinct expression signatures between Pcdh9^+^ fibroblasts and other subpopulations (Figure 4D; Figure S3A). Comparative analysis of DEGs between intervention groups (ICI, IR, iRT) and control group indicated pronounced transcriptomic alterations across all fibroblast subpopulations, most prominently in the iRT group versus Con group (Figure 4C; Figure S4A–F). GO and KEGG enrichment analyses of up‐regulated genes in fibroblasts highlighted biological processes and pathways strongly associated with fibrogenesis, including extracellular matrix (ECM) organization, ECM structural constituent, ECM receptor interaction, collagen fiber organization, growth factor binding, and cell adhesion molecule binding (Figure S5B). Subtype‐specific KEGG analysis further indicated that Pcdh9^+^ fibroblasts are particularly involved in cell adhesion and junction assembly, suggesting a role in structural remodeling (Figure S5A).

To further elucidate how radioimmunotherapy modulates the pro‐fibrotic and pro‐inflammatory roles of fibroblasts, we next compiled a customized gene set encompassing key profibrotic mediators, including cytokines, chemokines, growth factors, MMPs, and ECM markers [14, 15, 16, 17, 18, 19]. First, comparative analysis of fibrotic molecules across groups demonstrated that fibroblasts in the intervention groups, particularly the iRT group, exhibited robustly elevated expression of ECM components relative to control group (Figure 4E; Figure S5C). Furthermore, the iRT intervention group exhibited pronounced enhancement in various pro‐fibrotic factors, including *Tgfb1, Tgfb2, Pdgfa, Fgf2, Ccn2, Egf, Igf1, Serpine1, Vegfa, Lif, Il6, Il11, Il33, Cxcl1, Cxcl5, Cxcl10, Cxcl12, Ccl2, Ccl8, Ccl19*, along with fibrosis degradation factors involved *Mmp2, Mmp3, Mmp11, Mmp14, and Mmp19* (Figure 4F). Collectively, these findings indicate that fibroblasts acquire a markedly pro‐fibrotic phenotype following the combined treatment.

We further characterized the expression levels of ECM and fibrosis‐related molecules across fibroblast subtypes in the iRT group. While all fibroblast subpopulations shared similar ECM molecules expression patterns, quantitative differences were evident among subtypes (Figure S5D). Additionally, pro‐fibrotic factors, including growth factors (*Tgfb1, Tgfb2, Fgf2, Ccn2, Igf1, Serpine1, Vegfa*), chemokines (*Cxcl1, Cxcl10, Cxcl12, Ccl2, Ccl19*), and cytokines (*Il6, Il33*), were highly expressed across the seven fibroblast subpopulations. Although these factors were detectable in all subpopulations, their expression levels varied considerably, revealing a complex and heterogeneous pro‐fibrotic molecular network. Concurrent upregulation of *Mmp2, Mmp3, Mmp14, and Mmp19* suggested the concomitant activation of ECM degradation processes (Figure S5E). To elucidate the mechanisms underlying fibrosis initiation under combined radioimmunotherapy and identify key cardiac cell types driving this process, we compiled a set of profibrotic ligands and receptors pairs (Table S3) to clarify how different cell populations contribute to fibroblast activation. Cell‐cell interaction analysis revealed that within cardiac tissues of the iRT group, multiple profibrotic ligand‐receptor pairs, particularly including TGFB_TGFBR, PDGFA_PDGFRA, IGF1_IGF1R, and FGF1_FGFR1, were markedly upregulated between fibroblasts and other cell types (Figure 4G). In addition to contributions from fibroblasts themselves and ECs, immune cells were identified as important contributors to fibroblast activation (Figure 4G). We therefore further evaluated the potential impacts of fibroblasts on immune cells in the iRT group. Analysis of chemokine ligand‐receptor interactions indicated that fibroblasts primarily modulate immune cells through two key signaling axes: CXCL12/CXCL14_CXCR4 and CCL2_CCR2 (Figure 4H). Chord diagram analysis of signaling pathway interactions further indicated that fibroblasts and immune cells act as dominant signal senders within CCL and CXCL pathways, while fibroblasts and vascular ECs serve as primary signal receivers in the VCAM pathway (Figure 4I). These findings suggest that fibroblasts may play a critical role in orchestrating immune cell recruitment and infiltration into myocardial tissue, providing new mechanistic insights into the increased immune infiltration observed in hearts subjected to combined radioimmunotherapy.

### 2.5 The Increased Infiltrating Macrophages Demonstrated the Notable Effect in Promoting Fibroblast Activation in Cardiac Fibrosis Induced by CIR Combined with ICI

**FIGURE 5:**
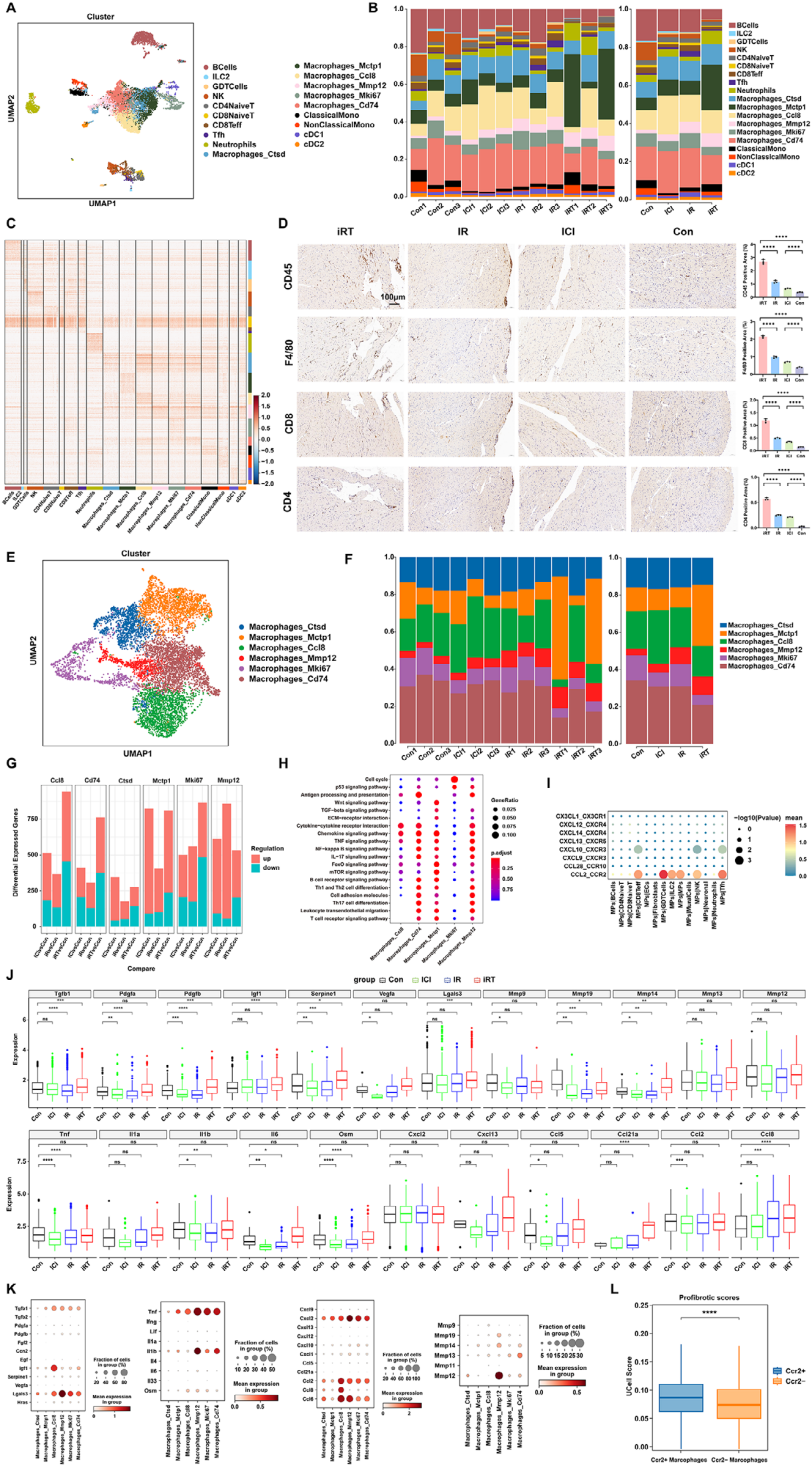
The increased infiltrating macrophages demonstrated a notable effect in promoting the activation of fibroblasts in cardiac fibrosis treated with ICI and CIR. (A) UMAP visualization of scRNA‐seq from immune cells in mouse hearts treated with ICI or/and CIR. (B) The proportion of immune cell subtypes in mice hearts across 12 mouse heart samples from Con, ICI, IR, and iRT groups (left), and after merging by intervention group (right). (C) Heatmap of the top 100 DEGs in each subset identified through unsupervised clustering of all the immune cells. Blue indicates lower expression, and red indicates higher expression. The expression scale is shown on the right. (D) Immune cell infiltration was analyzed by immunohistochemistry. CD45^+^ was used as a marker for leukocytes, F4/80^+^ for macrophages, and CD4^+^ and CD8^+^ were used to identify CD4^+^ T lymphocytes and CD8^+^ T lymphocytes, respectively, scale bar = 100 µm. (E) UMAP visualization of scRNA‐seq from macrophages in mouse hearts treated with ICI and/or CIR. (F) The proportion of macrophage subtypes in mice hearts across 12 mouse heart samples from Con, ICI, IR, and iRT groups (left), and after merging by intervention group (right). (G) The number of DEGs in macrophage subtypes of the mouse hearts for ICl vs Con, IR vs Con, and iRT vs Con. Red blocks represent upregulated genes, while teal blocks represent downregulated genes. (H) The KEGG enrichment analysis of upregulated genes of macrophage subtypes. (I) The chemokine interaction diagram of MPs acting as ligand cells with other cell types in the iRT group. (J) The box plots showing differential expression levels of pro‐fibrotic and fibrinolysis‐related factors in macrophages from cardiac tissues of Con, ICI, IR, and iRT groups. (K) The dot plots showing differential expression levels of pro‐fibrotic and fibrinolysis‐related factors across macrophage subtypes in the iRT group. (L) The box plots showing the expression scores of pro‐fibrotic gene sets in CCR2^+^ and CCR2^−^ macrophage clusters. Data are presented as mean ± SD, the one‐way ANOVA (D) were used to compare data. Data are presented as box plots showing the median, interquartile range, and potential outliers, differences between groups were assessed using the nonparametric Wilcoxon rank‐sum test (J, L). ns: not significant, *p < 0.05, **p < 0.01, ***p < 0.001, ****p < 0.0001.

Although immune cells constitute a relatively minor proportion of cardiac tissue, they play pivotal roles in cardiac growth and development, and the pathophysiology of cardiovascular diseases. To investigate the interaction mechanisms between fibroblasts and immune cells, we classified and characterized immune cell types using scRNA‐seq data. Within the cardiac immune microenvironment, we identified four major immune cell populations, including MPs, TandNK cells, B cells, and neutrophils, which were further subdivided into nineteen distinct subclusters (Figure 5A; Figure S6A). We further delineated the proportional distribution and transcriptional profiles of these immune subpopulations across samples (Figure 5B,C). Notably, compared to the Con group, the proportion of MPs within immune cells was significantly increased in the ICI, IR, and iRT groups, with the most prominent expansion observed in the iRT group (Figure S6B). Consistently, in the radioimmunotherapy‐induced mouse model, ICI or/and CIR, especially their combination, profoundly altered the infiltration patterns of T lymphocytes and macrophages (Figure 5D). To decipher the potential implications of increased MPs in the iRT group, we analyzed ligand–receptor interactions between MPs and other cell types. Chemokine signaling analysis revealed that MPs may modulate the activation and targeted migration of other immune cells primarily through the CXCL10_CXCR3 and CCL2_CCR2 pathways (Figure 5I).

Focusing specifically on the MPs population, we identified 8947 MPs through scRNA‐seq analysis and further classified them into five distinct subtypes based on transcriptional profiles: macrophages (8068 cells, predominant subtype), classical monocytes, non‐classical monocytes, conventional type 1 dendritic cells (cDC1), and cDC2 cells (Figure S6C). Within the macrophage populations, unsupervised clustering analysis of macrophage subsets revealed six transcriptionally distinct subpopulations with diverse biological characteristics (Figure 5E). Among them, the proportion of Mmp12^+^ and Mctp1^+^ macrophage subtypes were significantly increased following cardiac irradiation and ICI intervention (Figure 5F). To investigate the functional heterogeneity among these subsets, we performed KEGG pathway enrichment analysis utilizing upregulated genes specific to each subtype. The results indicated that both Mmp12^+^ and Mctp1^+^ macrophages were also significantly enriched in pathways related to inflammatory response, fibrotic formation, and immune cell differentiation (Figure 5G,H; Figures S3B and S7A–F).

To further elucidate the specific role of macrophages in fibroblast activation and fibrotic progression under combined radioimmunotherapy, we analyzed the expression patterns of pro‐fibrotic mediators across the four groups. Furthermore, comparative analysis of fibrosis‐related molecule expression in macrophages across the four experimental groups revealed significant upregulation of multiple pro‐fibrotic and inflammation‐related factors in the ICI, IR, and especially iRT groups compared to the control group, such as *Tgfb1, Pdgfa, Pdgfb, Igf1, Serpine1, Vegfa, Lgals3, Tnf, Il1b, Il6, Osm, Ccl2*, and *Ccl8* (Figure 5J). These findings indicate that macrophages exert a pronounced pro‐fibrotic effect when ICI combined with CIR. We further examined the expression and distribution of these pro‐fibrotic mediators across macrophage subtypes in iRT group. A range of key fibrogenic factors—including *Tnf, Il1b, Osm, Cxcl12, Cxcl10, Cxcl1, Ccl2, Ccl5, Ccl8, Tgfb1, Pdgfa, Pdgfb, Igf1, Serpine1*, and *Lgals3*—were highly expressed across the six macrophage subtypes, with particularly prominent levels observed in the Mmp12^+^ and Ccl8^+^ macrophage subpopulations (Figure 5K).

The CCR2_CCL2 axis plays a critical role in mediating immune cell migration and tissue infiltration. CCR2 acts as a navigational beacon by recognizing and binding to chemokines such as CCL2, thereby guiding immune cells to specific tissue compartments. Following ICI, IR, or iRT intervention, we observed a significant increase in the proportion of CCR2 positive expression (CCR2^+^) macrophages. Evaluation of profibrotic gene set scores revealed that these CCR2^+^ macrophages exhibited a more pronounced profibrotic phenotype compared to their CCR2^−^ macrophages (Figure 5L; Figure S6D–F).

### 2.6 The Characterization and Function of Lymphocytes in Myocardial Fibrosis Induced by CIR Combined with ICI

**FIGURE 6:**
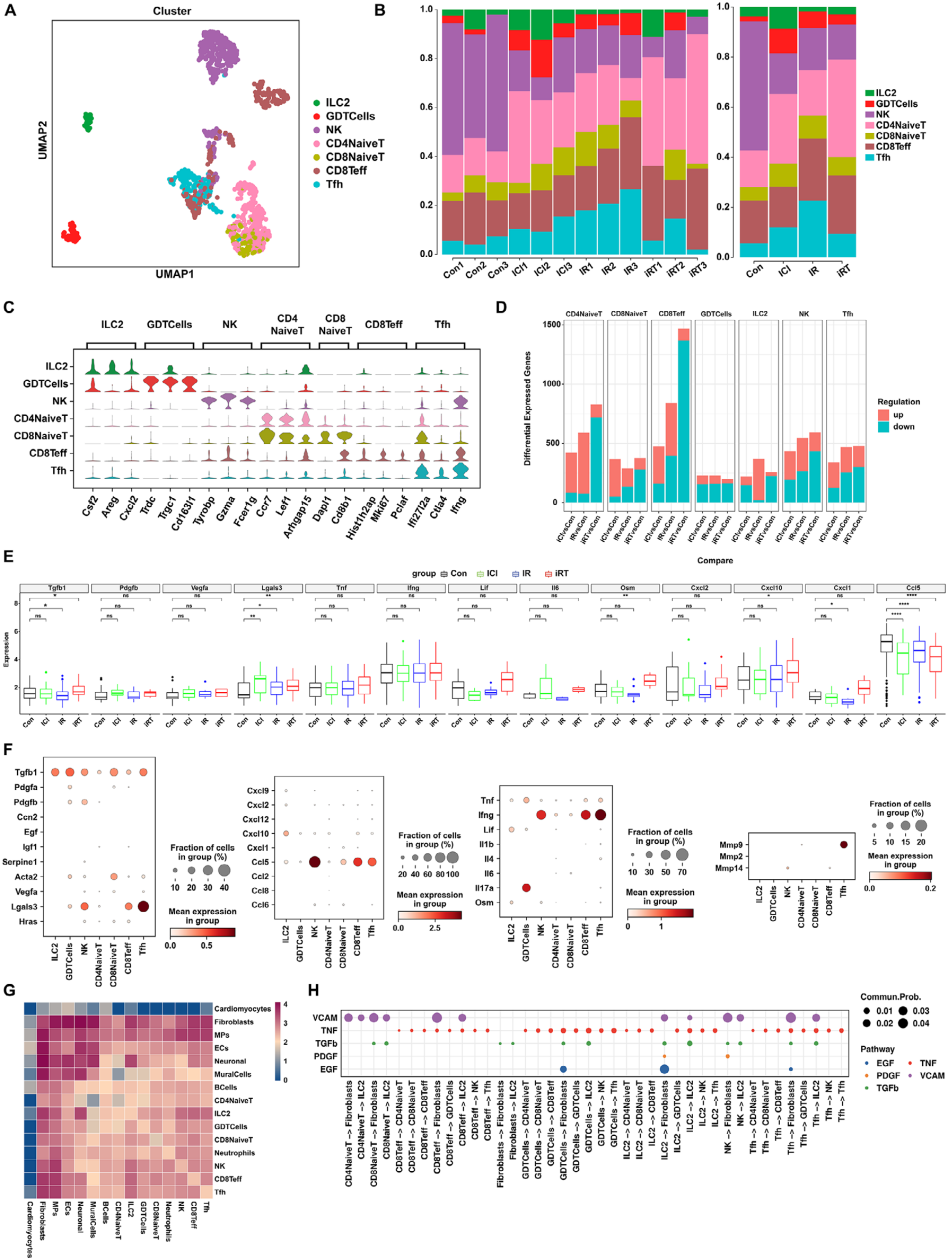
The identification and characterization of subpopulations of lymphocytes in cardiac fibrosis treated with ICI and CIR. (A) UMAP visualization of scRNA‐seq from lymphocytes in mouse hearts treated with ICI or/and CIR. (B) The proportion of lymphocytes subtypes in mice hearts across 12 mouse heart samples from Con, ICI, IR, and iRT groups (left), and after merging by intervention group (right). (C) Stacked violin plot of the top 3 marker genes for lymphocyte subtypes. (D) The number of DEGs in lymphocyte subtypes of the mouse hearts for ICl vs Con, IR vs Con, and iRT vs Con. Red blocks represent upregulated genes, while teal blocks represent downregulated genes. (E) The box plots showing differential expression levels of pro‐fibrotic and fibrinolysis‐related factors in lymphocytes from cardiac tissues of Con, ICI, IR, and iRT groups. (F) The dot plots showing differential expression levels of pro‐fibrotic and fibrinolysis‐related factors across lymphocyte subtypes in the iRT group. (G) Heatmap of cell‐cell interactions between lymphocyte subtypes and other cell types in iRT group. (H) The dot plots showing the cell‐cell interactions intensity of TGFb, VCAM, EGF, PDGF, and TNF signaling pathway network in the iRT group. Data are presented as box plots showing the median, interquartile range, and potential outliers. Differences between groups were assessed using the nonparametric Wilcoxon rank‐sum test (E). ns: not significant, *p < 0.05, **p < 0.01, ***p < 0.001, ****p < 0.0001.

We further investigated the role of lymphocytes in the cardiac fibrosis induced by combined radioimmunotherapy. By subclustering 1,228 TandNK cells, we identified and annotated seven distinct lymphocyte subsets, including group 2 innate lymphoid cells (ILC2), gamma delta T cells (GDTCells), natural killer cells (NK), CD4^+^ naive T cells (CD4NaiveT), CD8^+^ naive T cells (CD8NaiveT), CD8^+^ effector T cells (CD8Teff), and follicular helper T cells (Tfh) (Figure 6A). Subpopulation analysis revealed significant shifts in the proportions of specific T lymphocyte subsets when the intervention groups compared with the control group, including CD8Teff, Tfh, CD8 NaiveT, and CD4 NaiveT cells (Figure 6B). Notably, the combined intervention group exhibited a marked increase in CD8Teff and CD4NaiveT cells, whereas the proportion of NK cells was significantly reduced following treatment (Figure 6B). To decipher the functional alterations of lymphocyte subsets under different interventions, we compared transcriptional changes across the Con, ICI, IR, and iRT groups (Figure 6C; Figure S3C). DEG analysis between each intervention group (ICI, IR, iRT) and the control group revealed substantial transcriptomic reprogramming across lymphocyte subsets, with the iRT versus Con group having the largest number of DEGs in subsets of CD8Teff, CD4NaiveT, NK, and Tfh. (Figure 6D; Figure S8A–F).

We then evaluated the expression of fibrosis‐related activity molecules within these lymphocytes to explore their potential crosstalk with fibroblasts. Comparative analysis of fibrosis‐associated molecules across the four groups showed particularly enhanced expression of *Tgfb1, Lgals3, Osm*, and *Cxcl10* in lymphocytes from the iRT group versus Con group (Figure 6E). We also observed a series of key pro‐fibrotic mediators, including *Tgfb1, Pgdfb, Lgals3, Tnf, Ifng, Lif, Il17a, Ccl5*, and *Cxcl10*, were highly expressed in iRT group across the seven lymphocytes subsets, indicating active lymphocyte involvement in regulating a pro‐fibrotic molecular network under combined intervention (Figure 6F). Subsequent analysis revealed pronounced crosstalk between lymphocytes and stromal cells in iRT‐treated hearts, with interaction heatmaps demonstrating particularly robust crosstalk between TandNK cells and fibroblasts, MPs, and ECs (Figure 6G). Moreover, further analysis suggested that lymphocyte subsets may promote fibroblast activation and fibrotic processes through signaling pathways such as TGF‐β, VCAM, EGF, PDGF, and TNF under combined radioimmunotherapy (Figure 6H).

### 2.7 Comprehensive Single‐Cell Mapping of Immune Checkpoint Landscapes in the Myocardial Microenvironment after Radioimmunotherapy

Immune checkpoint molecules serve as critical regulators of antitumor immunity and the maintenance of self‐tolerance. However, their dynamic expression patterns within the cardiac immune microenvironment, particularly under combined radioimmunotherapy, remains poorly understood and warrants systematic investigation. To address this, we comprehensively evaluated the expression levels of key immune checkpoint molecules across all nine cell types in the Con, ICI, IR, and iRT groups (Figure S9A,B).

We first examined the expression of T‐cell co‐stimulatory molecules CD28 and CD86. The results showed a progressive increase in *Cd28* expression within TandNK cells from the control group to the intervention groups (ICI, IR, and iRT). Concurrently, *Cd86* expression was significantly upregulated in MPs from cardiac tissues subjected to combined radioimmunotherapy. These findings suggest that enhanced activation of the CD28/CD86 co‐stimulatory axis may contribute to excessive T cell activation under radioimmunotherapy conditions (Figure S9A,C,D). We next analyzed the transcriptional expression patterns of the immunosuppressive molecules PD‐1 (encoded by *Pdcd1*) and PD‐L1 (encoded by *Cd274*) in cardiac cells across the four groups. *Pdcd1* was predominantly expressed on immune cells across four groups, while *Cd274* was broadly distributed across multiple cell types. Pdcd1 molecules maintained relatively stable expression levels across all experimental groups in nine cardiac cell types (Figure S9A,E). In addition, neutrophils in the control group exhibited higher *Cd274* expression compared to the other groups, while *Cd274* levels remained consistently low across other cardiac cell types among four groups (Figure S9A,F). These observations indicate that, despite the dual intervention of ICI and irradiation, the PD‐1/PD‐L1 signaling pathway may not undergo significant transcriptional fluctuations in cardiac tissue.

### 2.8 Increased IL‐6 Expression in the Fibroblasts of Mice Hearts in Radioimmunotherapy‐Induced Cardiac Fibrosis, Positively Correlating with Resultant Inflammation and Fibrosis

**FIGURE 7:**
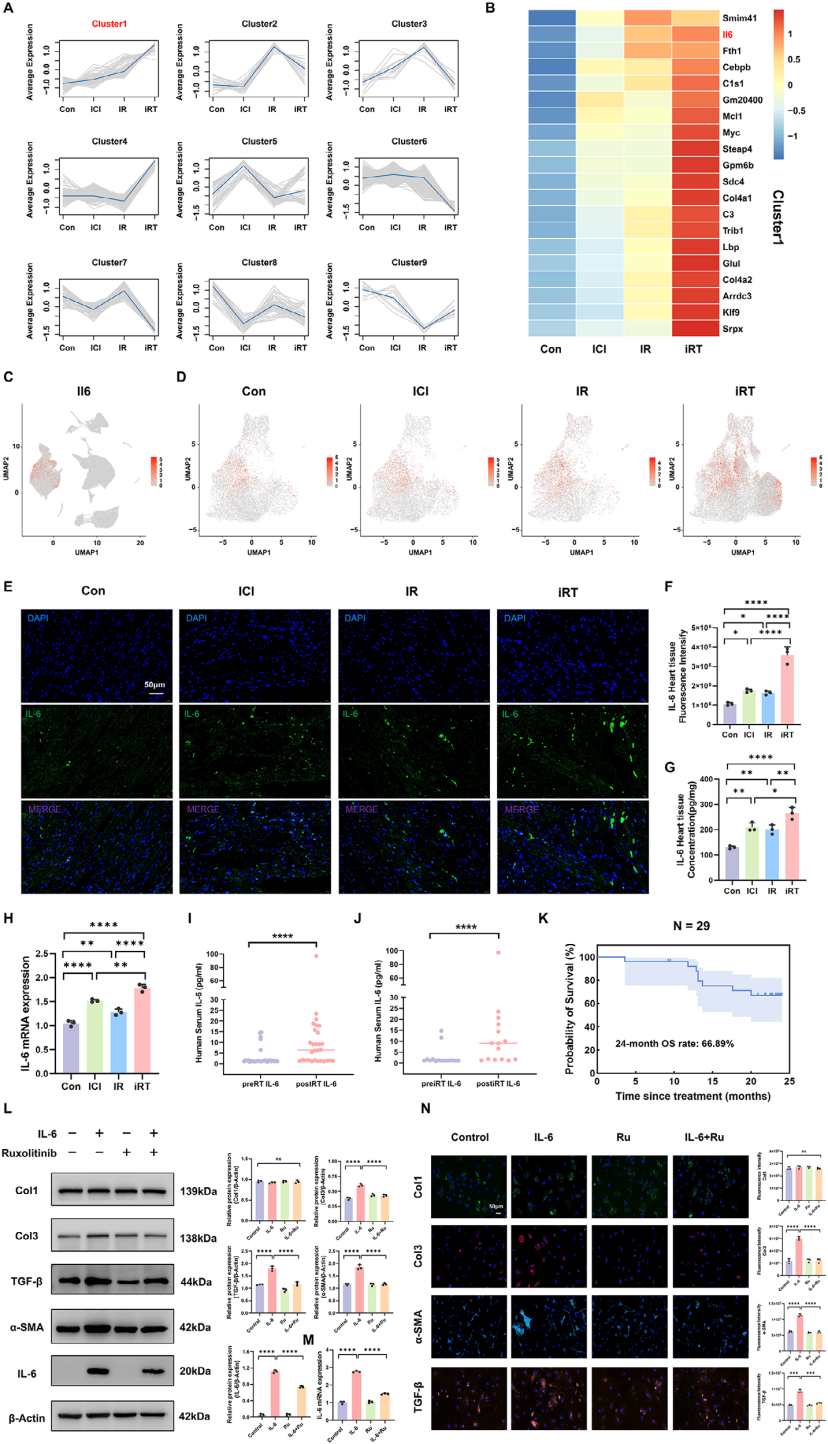
Increased IL‐6 expression in the fibroblasts of mice in radioimmunotherapy‐induced cardiac fibrosis. (A) Trend analysis of differential gene expression in fibroblasts from Con, ICI, IR, and iRT groups, categorized into nine distinct expression pattern clusters. (B) Heatmap of the expression levels of the top 20 genes in cluster 1 across the four groups. (C) Distribution and expression of IL‐6 across nine cardiac cell types visualized in feature plot. (D) Distribution and expression of IL‐6 in fibroblasts among Con, ICI, IR, iRT groups visualized in the feature plot. (E) Immunofluorescence analysis of IL‐6 expression in mice cardiac tissues from Con, ICI, IR, and iRT groups at day 28 post‐treatment, scale bar = 50 µm. (F) Quantification histogram of IL‐6 fluorescence intensity. (G) IL‐6 expression level of cardiac tissues in mice across four groups at day 28 post‐treatment detected by ELISA. (H) qRT‐PCR analysis of IL‐6 transcript levels in cardiac tissues from the four mouse groups at day 28 post‐treatment. (I) The post‐thoracic radiotherapy (post‐RT) and baseline (pre‐RT) of serum IL‐6 expression levels in 29 patients with malignant thoracic tumors were assessed by ELISA. (J) The post‐iRT(post‐iRT) and baseline (pre‐iRT) of serum IL‐6 expression levels in 15 patients with malignant thoracic tumors were assessed by ELISA. (K) Kaplan‐Meier curve showing overall survival in the cohort of 29 thoracic tumor patients. (L) Western blot analysis of Col1, Col3, α‐SMA, TGF‐β, and IL‐6 protein levels in primary mouse fibroblasts stimulated by murine IL‐6 cytokine with or without ruxolitinib for 48 h; quantitative analysis of protein expression levels. (M) qRT‐PCR analysis of IL‐6 transcript levels in primary mouse fibroblasts stimulated by murine IL‐6 cytokine with or without ruxolitinib for 48 h. (N) IF staining of α‐SMA, TGF‐β, Col1, and Col3 in primary mouse fibroblasts stimulated by murine IL‐6 cytokine with or without ruxolitinib for 48 h, scale bar = 50 µm; quantitative histograms of corresponding markers. Data are presented as mean ± SD. The Kaplan‒Meier method was used to assess patient survival (K). Differences between groups were assessed using the one‐way ANOVA (F, G, H, L, M, N) and the Wilcoxon signed‐rank test (I,J). ns: not significant, *p < 0.05, **p < 0.01, ***p < 0.001, ****p < 0.0001.

In investigating the mechanisms of cardiac fibrosis induced by combined CIR and ICI, our scRNA‐seq analysis revealed complex interaction patterns between cardiac fibroblasts and immune cells under combined therapy. To decipher the crucial molecular mechanisms driving this pathological process, we performed a systematic expression trends analysis of DEG in cardiac fibroblasts across Con, ICI, IR, and iRT group. Based on transcriptional profiles of fibroblasts from these groups, we identified nine distinct gene clusters exhibiting unique expression dynamics. Notably, genes in Cluster1 demonstrated a significant and sustained upward trend, with expression levels progressively increasing from the Con group to the ICI, IR, and iRT groups (Figure 7A). Among the top 30 key genes in Cluster1, we specifically identified IL‐6 as a molecule of interest (Figure 7B; and Table S4). We also identified that an overwhelming proportion of IL‐6 positive expression cardiac cells across the four groups are derived from cardiac fibroblasts, with the proportion ranging from 86.3% to 89.5% (Figure S10A and Table S5). Further visualization of IL‐6 expression distribution via UMAP plots confirmed its specific enrichment in fibroblasts across all cardiac cell types, and showed that both expression level and distribution of IL‐6 in fibroblasts increased stepwise across treatment groups from control to ICI, IR, and iRT (Figure 7C,D; Figure S10B). Based on this expression pattern, we defined IL‐6^+^ fibroblasts as the subset of cardiac fibroblasts exhibiting IL‐6 positive expression (see the Methods in the Supporting Information). Interestingly, we also found that these Cluster 1 genes were broadly and highly enriched in IL‐6^+^ fibroblasts within the iRT group (Figure S10C,D). We also employed ELISA, qRT‐PCR, and IF staining to assess IL‐6 expression in cardiac tissues following combined intervention. Results consistently showed a significant increase in IL‐6 expression in cardiac tissues from the iRT group compared to Con, ICI, and IR groups at day 28 post intervention (Figure 7E–H). Furthermore, the protein expression level of IL‐6 in the iRT group remained elevated at 3‐ and 5‐ months post‐intervention compared to the other groups (Figure S10E). To extend these observations to a clinical context, we prospectively collected serum samples from 29 patients with thoracic malignancies before and after thoracic radiotherapy. Serum analysis demonstrated a significant elevation of IL‐6 levels after thoracic radiotherapy compared with baseline (*p* < 0.0001). Among these, 15 patients who received combined radiotherapy and immunotherapy also exhibited significantly elevated serum IL‐6 levels following treatment compared to pre‐treatment baseline (*p* < 0.0001) (Figure 7I,J; and Table S6). In this cohort of 29 patients with thoracic malignancies, the estimated 24‐month OS rate was 66.89% (Figure 7K). To further validate these findings in vitro, primary fibroblasts were treated with 0 or 100 ng/mL of IL‐6 for 48 h. Subsequent analysis via Western blot and qRT‐PCR showed that IL‐6 stimulation significantly upregulated both IL‐6 protein and mRNA expression in fibroblasts (Figure 7L,M). Furthermore, elevated protein and mRNA expression levels of Col3, TGF‐β, and α‐SMA were observed, while Col1 expression remained relatively unchanged (Figure 7L,N; Figure S10G). Notably, IL‐6 stimulation also consistently increased the protein levels of JAK2, phosphorylated JAK2 (p‐JAK2), STAT3, and p‐STAT3 in fibroblasts (Figure S10F). To directly validate the involvement of the JAK2/STAT3 pathway, we have also administrated the specific JAK1/2 inhibitor Ruxolitinib to pre‐treated cardiac fibroblasts with 1 µm prior to IL‐6 stimulation. The results indicated that ruxolitinib abolished IL‐6‐induced phosphorylation of JAK2 and STAT3, and significantly attenuated the upregulation of fibrotic markers including α‐SMA, TGF‐β and Col3 (Figure 7L–N; Figure S10F,G), conclusively demonstrate that IL‐6 exerts its pro‐fibrotic effect primarily through the JAK2/STAT3 signaling pathway.

### 2.9 Central Role of IL‐6^+^ Fibroblast‐Macrophage Crosstalk in Radioimmunotherapy‐Induced Cardiac Fibrosis

**FIGURE 8:**
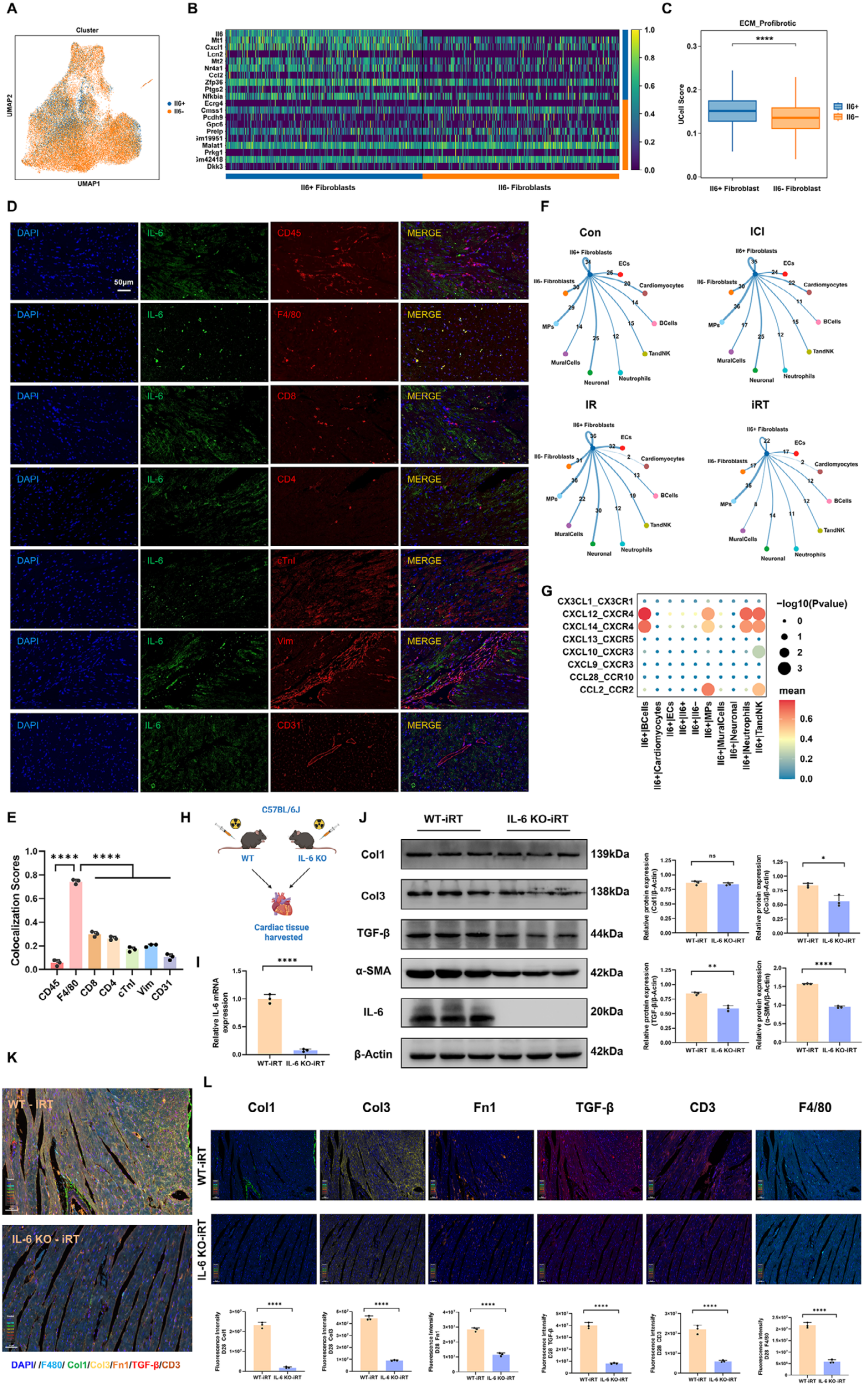
IL‐6 positive fibroblasts involved in promoting cardiac inflammation and fibrosis. (A) UMAP visualization of scRNA‐seq from IL‐6^+^ and IL‐6^−^ fibroblasts in mouse hearts treated with ICI or/and CIR. (B) Heatmap of the top 20 DEGs between IL‐6^+^ and IL‐6^−^ fibroblasts identified through unsupervised clustering. Blue indicates lower expression, and yellow indicates higher expression. The expression scale is shown on the right. (C) The box plots showing the expression scores of pro‐fibrotic and ECM gene sets in IL‐6^+^ and IL‐6^−^ fibroblast clusters. (D) The immunofluorescence co‐localization analysis of inflammatory factor IL‐6 with leukocytes (CD45^+^), macrophages (F4/80^+^), CD8^+^ T cells, CD4^+^ T cells, cardiomyocytes (cTnI^+^), fibroblasts (Vim^+^), and endothelial cells (CD31^+^), scale bar = 50 µm; (E) The statistical column plots represent the comparison of colocalization scores among various indicators. (F) Network diagram depicting the interaction frequency between IL‐6^+^ fibroblasts and other cardiac cell types in Con, ICI, IR, and iRT groups. Line width represents the number of the ligand–receptor pairs. (G) The chemokine interaction diagram of IL‐6^+^ fibroblasts acting as ligand cells with other cell types in the iRT group. (H) Schematic of the experimental design for radioimmunotherapy in IL‐6 wild‐type (WT) and IL‐6 knockout (KO) mice (*n* = 4 per group). (I) qRT‐PCR analysis of IL‐6 transcript levels in cardiac tissues from WT and IL‐6 KO mice. (J) Western blot analysis of Col1, Col3, TGF‐β, α‐SMA, and IL‐6 protein expression in hearts of WT and IL‐6 KO mice treated with radioimmunotherapy; quantitative analysis of protein expression levels (*n* = 3 per group). (K) Representative multiplex immunofluorescence images of cardiac tissues from WT and IL‐6 KO mice after combined radioimmunotherapy, scale bar = 50 µm. (L) Single‐channel immunofluorescence validation and quantitative analysis of the markers in cardiac tissues from WT and IL‐6 KO mice after combined radioimmunotherapy. Staining markers: DAPI (nuclei, blue), Col1 (green), Col3 (yellow), Fibronectin 1 (orange), TGF‐β (red), CD3 (T cells, brown), F4/80 (macrophages, light blue), scale bar = 50 µm; the corresponding quantitative histograms of each marker are shown. Data are presented as mean ± SD. The unpaired t‐test (I, J, L) and the one‐way ANOVA (E) was performed to compare data. Data are presented as box plots showing the median, interquartile range, and potential outliers, differences between groups were assessed using the nonparametric Wilcoxon rank‐sum test (C). ns: not significant, *p < 0.05, **p < 0.01, ***p < 0.001, ****p < 0.0001.

To investigate the functional specificity of IL‐6^+^ fibroblasts, we stratified fibroblasts into IL‐6^+^ and IL‐6^−^ subpopulations and analyzed their transcriptional profiles (Figure 8A). Profibrotic gene set scoring revealed that IL‐6^+^ fibroblasts exhibited a significantly stronger profibrotic signature compared to IL‐6^−^ fibroblasts (Figure 8B,C). Consistent with this, GO enrichment analysis of up‐regulated DEGs in IL‐6^+^ fibroblasts demonstrated significant enrichment in biological processes closely associated with pro‐inflammatory and profibrotic responses, including leukocyte and mononuclear cell differentiation, chemokine and cytokine activity, and regulation of smooth muscle cell proliferation (Figure S11A). These findings were corroborated by KEGG pathway analysis, which indicated prominent enrichment of IL‐6^+^ fibroblasts in key inflammatory signaling pathways such as TNFα, NF‐kappaB, and IL‐17 (Figure S11B). To further delineate the interactions between IL‐6^+^ fibroblasts and other cell types, we performed a comprehensive cell‐cell communication analysis. Quantitative assessment of interaction frequency revealed that among all four groups, IL‐6^+^ fibroblasts interacted most frequently with MPs (Figure 8F). Further focused analysis of the iRT group highlighted the CXCL12/CXCL14_CXCR4 and CCL2_CCR2 chemokine signaling axes as central mediators of communication between IL‐6^+^ fibroblasts and immune cells, including B cells, MPs, neutrophils, and TandNK cells, which may suggest a potential role for these fibroblasts in promoting immune cell recruitment and infiltration (Figure 8G). Remarkably, the interaction chord diagram visualization of IL‐6 signaling in the iRT group further highlighted the particularly close communication between IL‐6^+^ fibroblasts and MPs (Figure S11C). Immunofluorescence co‐localization further supported these findings, showing robust co‐localization of IL‐6 with macrophages (F4/80^+^) in cardiac tissues following combined radioimmunotherapy, whereas its association with T cells (CD4^+^, CD8^+^), fibroblasts (Vimentin^+^), cardiomyocytes (cTnI^+^), or endothelial cells (CD31^+^) was comparatively weak (Figure 8D,E). These results suggest a close functional relationship between IL‐6 secretion and fibroblast‐macrophage crosstalk in the context of radioimmunotherapy‐induced cardiac fibrosis.

Further analysis of the expression pattern of IL‐6 receptor (IL‐6RA) across different cardiac cell types revealed its predominant enrichment in MPs (Figure S12A,B). Although widely distributed across various macrophage subtypes, no exceptionally high or subtype‐specific expression of IL‐6R was observed within individual macrophage subsets (Figure S12C). Given the multifaceted role of macrophages in modulating immune cell activity and maintaining cardiac microenvironment homeostasis, we next investigated their responsiveness to IL‐6 signaling pathway. To delineate the specific regulatory impact of IL‐6^+^ fibroblasts on macrophages, we scored IL‐6 signaling activity and stratified macrophages into two subgroups based on high or low pathway activity (Figure S12D,E). Functional characterization showed that macrophages with high IL‐6 signaling activity exhibited markedly elevated expression of the T cell co‐stimulatory antigen CD86, along with reduced transcriptional levels of PD‐1 and PD‐L1 (Figure S12F). Notably, however, they were significantly enriched in the PD‐L1 expression and PD‐1 checkpoint pathway (Figure S12G). This apparent discrepancy arises because the pathway activity score reflects not only receptor and ligand expression but also downstream signaling events, particularly the JAK‐STAT signal cascade, that are robustly upregulated upon IL‐6 stimulation (Table S7). Consequently, even when PD‐1/PD‐L1 transcript levels are modestly reduced, IL‐6 may enhance the functional efficacy of PD‐1/PD‐L1 signaling through intensified JAK‐STAT pathway activity, thereby reconciling the divergence between surface marker expression and overall pathway activation. To directly validate the impact of IL‐6 on macrophage function, we exogenously treated RAW264.7 macrophages with bioactive IL‐6 cytokine. Stimulation with 50 ng/mL IL‐6 resulted in a significant increase in CD86 expression on macrophages surface (Figure S12H). These finding provides functional insight into the role of IL‐6 in modulating macrophage functional states and checkpoint molecule expression.

### 2.10 IL‐6 Deficiency Attenuate Cardiac Injury and Decreases Cardiac Fibrosis

To elucidate the specific role of IL‐6 in cardiac fibrosis induced by combined radioimmunotherapy, we first established a murine model of IL‐6 KO mice subjected to combined treatment, aiming to investigate the impact of IL‐6 deficiency on fibrotic progression (Figure 8H,I). Multiplex immunofluorescence staining and Western blot analysis revealed that, compared to WT mice, IL‐6 KO mice receiving combined intervention exhibited significantly reduced ECM deposition and immune cell infiltration in cardiac tissues (Figure 8J–L). We also observed that, compared to WT mice, IL‐6 KO mice subjected to combined radioimmunotherapy showed no significant change in total JAK2 and STAT3 protein levels, but exhibited a marked reduction in p‐JAK2 and p‐STAT3 (Figure S11D). In addition, we analyzed liver and kidney function in the serum of IL‐6 KO and WT mice subjected to radioimmunotherapy, demonstrating that IL‐6 KO did not cause significant impairment of hepatic or renal function (Supporting Information).

**FIGURE 9:**
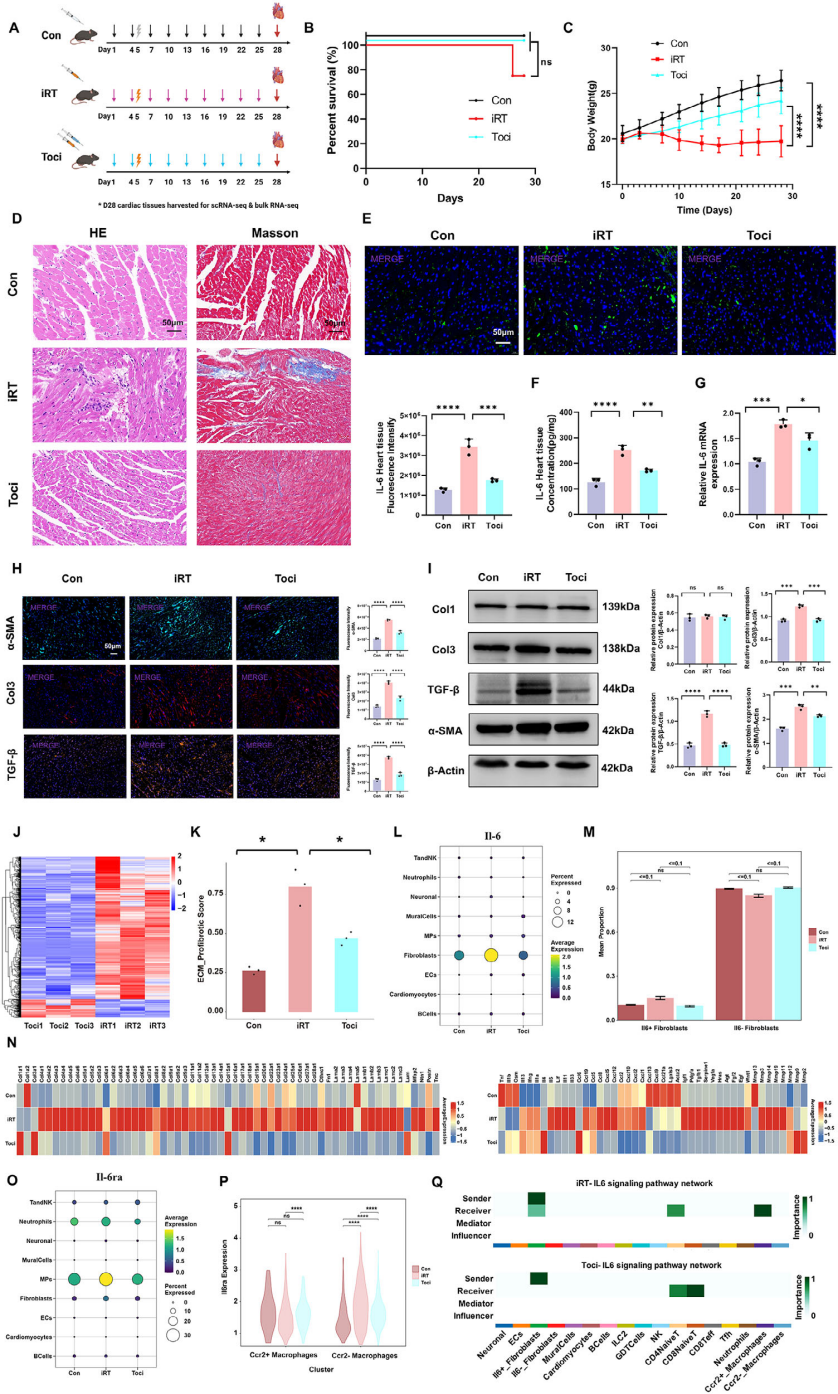
Tocilizumab attenuates radioimmunotherapy‐induced cardiac injury and fibrosis via the bulk and single‐cell transcriptomic profiling. (A) Schematic of the of IL‐6 receptor (IL‐6 RA) inhibitor tocilizumab intervention in radioimmunotherapy‐induced cardiac injury mouse model. (B) Survival curves of mice over day 28 in Con, iRT, and Toci groups. Data are presented as Kaplan‐Meier survival curves, statistical analysis was performed using the Log‐rank test (*n* = 8 per group). No significant difference in survival rate was observed among the three groups (*p* = 0.14). Two cases of acute death occurred in the iRT group, while no mortality was detected in the control and Toci groups. (C) Body weight changes of mice in Con, iRT, and Toci groups at day 28 (*n* = 6 per group). (D) HE and Masson's trichrome staining for estimating cardiac injury and fibrosis in Con, iRT, and Toci mice cardiac tissue at day 28 post‐treatment, scale bar = 50 µm. (E) Immunofluorescence analysis of IL‐6 expression levels in mice myocardial tissues from Con, iRT, and Toci groups, scale bar = 50 µm; quantification histogram of IL‐6 immunofluorescence intensity. (F) Cardiac tissue IL‐6 expression levels detected by ELISA in mice from Con, iRT, and Toci groups at day 28 (*n* = 3 per group). (G) qRT‐PCR analysis of IL‐6 transcript levels in cardiac tissues from Con, iRT, and Toci groups at day 28 post‐treatment (*n* = 3 per group). (H) Immunofluorescence staining of α‐SMA, TGF‐β, Col1, and Col3 in cardiac tissues of Con, iRT, and Toci groups at day 28 post‐intervention, scale bar = 50 µm; quantitative histograms of corresponding markers. (I) Western blot analysis of Col1, Col3, α‐SMA, and TGF‐β protein levels in myocardial tissues from Con, iRT, and Toci groups at day 28 post‐intervention; quantitative analysis of protein expression levels (*n* = 3 per group). (J) Heatmap of the DEGs in cardiac tissues from Toci vs iRT groups based on bulk transcriptome analysis (*n* = 3 per group). Blue indicates lower expression, and red indicates higher expression. The expression scale is shown on the right. (K) Expression scores of profibrotic and ECM gene sets from bulk transcriptomic analysis in Con, iRT, and Toci groups at day 28. (L) The dot plot showing IL‐6 expression levels across nine cardiac cell types in Con, iRT, and Toci groups (*n* = 3 per group) based on scRNA‐seq. (M) Proportion analysis of IL‐6^+^ and IL‐6^−^ fibroblasts in Con, iRT, and Toci groups from scRNA‐seq data. (N) The heatmap displaying expression levels of ECM molecules (left) and profibrotic/fibrinolysis‐related factors (right) in fibroblasts across the three groups based on scRNA‐seq. (O) The dot plot showing IL‐6RA expression across nine cardiac cell types in Con, iRT, and Toci groups based on scRNA‐seq. (P) Proportion analysis of CCR2^+^ and CCR2^−^ macrophages in cardiac tissues across the three groups based on scRNA‐seq. (Q) Heatmap of IL‐6 signaling pathway interactions among cardiac cell types in iRT (upper) and Toci (lower) groups based on scRNA‐seq. Data are presented as mean ± SD. Differences between groups were assessed using the one‐way ANOVA (E, F, G, H, I, K), and the nonparametric Wilcoxon rank‐sum test (M, P). ns: not significant, *p < 0.05, **p < 0.01, ***p < 0.001, ****p < 0.0001.

To further validate the pivotal role of IL‐6 in regulating cardiac fibrosis, we administered tocilizumab, an IL‐6 receptor inhibitor, to evaluate its therapeutic efficacy in attenuating iRT‐induced myocardial fibrosis (Figure 9A). Log‐rank test showed no statistically significant difference (*p* = 0.14) in survival rates among the three groups (Figure 9B). Notably, two mice in the iRT group experienced acute death during the 28 days post‐intervention, whereas no mortality was recorded in the control group and tocilizumab (Toci) group. The trend was consistent with our preliminary observation, which may be attributed to the limited sample size in the current study. At the day 28 post‐intervention, compared to the iRT group, mice treated with tocilizumab demonstrated favorable weight gain, and exhibited significant reductions in serum biomarkers including cTnI and NT‐proBNP. Moreover, cardiac IL‐6 levels were markedly decreased, accompanied by attenuated cardiac dysfunction, and significant amelioration of cardiac inflammation, and fibrosis (Figure 9C–G; Figure S13A–C). In addition, Western blot and immunofluorescence analyses revealed a marked reduction of α‐SMA, TGF‐β, and Col3 expression in the Toci group compared to the iRT group (Figure 9H,I), and accompanied by significant decrease in JAK2/STAT3 pathway protein expression levels (Figure S13D). Notably, we extended our investigation to female mouse models undergoing combined radioimmunotherapy followed by Tocilizumab intervention (Figure S1A). We observed that tocilizumab treatment conferred a consistent protective effect against cardiac injury and fibrosis in both male and female mice following combined radioimmunotherapy (Figure S1B–H).

Bulk transcriptomic profiling of cardiac tissues from the Toci, iRT, and Con groups further supported these findings. Relative to the iRT group, the tocilizumab group displayed 218 significantly upregulated and 1722 significantly downregulated genes (Figure 9J). Profibrotic and ECM‐related gene set scoring demonstrated significantly lower fibrosis activity in the tocilizumab group than in the iRT group (Figure 9K). GSEA indicated pronounced downregulation in key pathways such as acute inflammatory response and acute phase response in tocilizumab‐treated mice compared to the iRT group (Figure S13E). Similarly, GO enrichment analysis showed decreased activity in processes including leukocyte migration, leukocyte chemotaxis, regulation of immune effector process, regulation of inflammatory response, cytokine‐mediated signaling pathway, collagen‐containing ECM, and collagen trimer formation in the Toci versus iRT group. In contrast, processes related to positive regulation of catabolic processes, positive regulation of cellular catabolic processes, contractile fiber organization, and myofibril assembly were upregulated (Figure S13F). Overall, IL‐6/IL‐6R inhibition effectively mitigates iRT‐induced cardiac dysfunction and myocardial fibrosis. To validate our findings in a tumor‐bearing context and assess the impact of tocilizumab on antitumor efficacy, we established an Lewis lung carcinoma (LLC) subcutaneous tumor‐bearing model and treated with the radioimmunotherapy and tocilizumab. Bioluminescence imaging, tumor fluorescence intensity analysis, and end‐point tumor volume measurement collectively revealed a significant reduction in tumor volume in the Toci group compared to the iRT group (Figure S14A–C). Furthermore, in this oncological setting, tocilizumab intervention effectively alleviated radioimmunotherapy‐induced cardiac fibrosis and inhibited activation of the JAK2/STAT3 pathway (Figure S14D–F). These results suggest that adjunctive tocilizumab not only mitigates cardiac toxicity but may also enhance the antitumor efficacy of radioimmunotherapy.

We further performed scRNA‐seq on cardiac tissues from mice treated with combined radioimmunotherapy followed by tocilizumab intervention. Consistent with previous findings, broad cell type classification showed a decreased proportion of fibroblasts in the Toci group compared to the iRT group (Figure S15A). Within the cardiac immune microenvironment, macrophages as well as ILC2, GDTCells, CD4^+^ naive T, CD8Teff, and Tfh cell populations were reduced among MPs as well as TandNK cells following tocilizumab treatment (Figure S15B–E). Although NK cells exhibited a relative elevation, this trend aligned with the overall changes across the Con, ICI, IR, and iRT groups (Figure S15B,C). Notably, IL‐6 continued to show specifically significant enrichment in fibroblasts across the Con, iRT, and Toci groups. Furthermore, both the proportion and expression level of IL‐6^+^ fibroblasts were markedly decreased after tocilizumab administration (Figure 9L,M). Further analysis revealed broadly significant downregulation of ECM components and pro‐fibrotic mediators in fibroblasts from the Toci group compared to the iRT group (Figure 9N). We also evaluated the expression and distribution of IL‐6RA across cell types. Although neutrophils also exhibited a relatively high abundance of IL‐6RA expression, their overall cell proportion in total cardiac tissue was minimal, and no significant intergroup differences in IL‐6RA expression were observed. IL‐6RA was most highly enriched in MPs and exhibited notably high expression levels, which were decreased following tocilizumab intervention (Figure 9O). We also observed that tocilizumab treatment led to a marked reduction of CD86 expression on macrophages with high IL‐6 signaling activity compared to radioimmunotherapy intervention (Figure S15F). Additionally, both the IL‐6RA expression of CCR2^+^ and CCR2^−^ macrophage subsets were significantly reduced in the Toci group compared to the iRT group (Figure 9P). Importantly, tocilizumab treatment substantially suppressed the IL‐6‐mediated activation signaling between IL‐6^+^ fibroblasts and macrophages, particularly CCR2^+^ macrophages, further suggesting its therapeutic effect in modulating pro‐fibrotic intercellular crosstalk in cardiac immune microenvironment (Figure 9Q).

## 3 Discussion

In the current landscape of clinical oncology, the combination of thoracic radiotherapy and ICI has exhibited robust antitumor efficacy. However, safety concerns regarding adverse reactions (ARs) have been increasingly brought to the forefront. Cardiac toxicity, although occurring less frequently, represents a substantial hazard due to its association with high mortality rates. The primary challenge for clinicians lies in optimizing the antitumor therapeutic effectiveness while effectively reducing or eliminating adverse reactions. Based on initial research, we identified increased cardiac toxicity risks from such combined treatment [5]. Utilizing preclinical models, we uncovered the exacerbating effect of this combined application on cardiac fibrosis. Through scRNA‐seq, we delved into the microscopic landscape of radioimmunotherapy‐induced cardiac injury, revealing fibroblast activation and significant breadth and intensity of interactions with cardiac immune cells. Notably, IL‐6 signaling in fibroblasts surged, intensifying with combined intervention. In IL‐6 knockout mice, inflammation and fibrosis were attenuated under the combined intervention. Furthermore, tocilizumab, an IL‐6 receptor inhibitor, alleviated early cardiac damage and fibrosis, suppressed the IL‐6‐mediated activation signaling on IL‐6^+^ fibroblasts and CCR2^+^ macrophages, offering a novel therapeutic avenue for mitigating radioimmunotherapy‐related cardiac toxicities.

The formation of local fibrotic scar tissue is an adaptive mechanism to preserve cardiac structural integrity and respond to inflammatory stimuli. However, chronic activation drives excessive fibrosis, characterized by reduced ventricular compliance, impaired diastolic function, inefficient contraction, diminished cardiac output, and disrupted electrical conduction [20]. Cardiac fibroblasts, residing in the interstitium, are central to remodeling owing to their capacity to synthesize and degrade ECM proteins [21]. In our scRNA‐seq analysis, fibroblasts accounted for 38.15% of cardiac cells, with proportions of 33.48% in the Con, 38.28% in ICI, 33.50% in IR, and 41.84% in iRT group. These values fall within the ranges reported in previous studies, where fibroblasts constitute 20–50% of cardiac cells and cardiomyocytes account for only 25–40% [22, 23]. The variability across studies largely reflects differences in tissue dissociation and scRNA‐seq capture efficiency, particularly given the low capture of large cardiomyocytes [24]. Our scRNA‐seq analysis also revealed marked fibroblast proliferation and activation, accompanied by elevated ECM molecules and fibrosis‐related markers. Consistent with previous work showing fibroblasts can self‐activating via TGF‐β and promote macrophage pro‐inflammatory activity via producing TGFβ [25, 26]. We also further observed that under combined intervention, fibroblasts displayed the enrichment of chemokines and inflammatory mediators including CXCL12, CXCL14, CCL2, and IL‐6, and actively modulated immune cell activity, suggesting they acquire immunomodulatory functions reminiscent of immune cells. These findings underscore the pivotal importance of fibroblasts, mediated through cytokines, growth factors, chemokines, and MMPs, in modulating fibroblast behavior during tissue injury and fibrotic progression.

Our scRNA‐seq analysis revealed increased infiltration of monocytes/macrophages, CD4 naive T cells, and CD8 Teff cells in cardiac tissues following combined intervention, consistent with our animal model findings and underscoring the central role of the immune microenvironment in early cardiac injury. ICI‐induced cardiotoxicity primarily arises from immune‐inflammatory activation [23], whereas radiation contributes through primarily cytotoxicity and immune modulation [27]. Consequently, in assessing the early risk of cardiac injury linked to their concurrent use, inflammatory cell infiltration and inflammatory cascade effect emerge as a key aspect in the development of cardiac harm [12]. Beyond increased activated CD8^+^ T lymphocyte can induce myocardial injury, there has been considerable debate on whether ICI exacerbate pathological T lymphocyte autoreactivity by promoting upregulated PD‐L1 expression in the myocardium. Interestingly, our scRNA‐seq analysis revealed that, except for neutrophils, the mRNA expression of PD‐1 and PD‐L1 was relatively stable in all cell components of the four groups, suggesting that cardiac PD‐L1/PD‐1 expression may not be significantly altered by ICI, CIR, or their combination. Notably, neutrophils in the control group exhibited higher baseline PD‐L1 expression compared to other groups, a finding potentially attributable to their natural immunoregulatory role in maintaining homeostasis, where constitutive PD‐L1 expression may restrain excessive immune activation under physiological conditions. The decreased PD‐L1 expression could represent either neutrophil exhaustion or a therapy‐induced shift toward a more pro‐inflammatory and immune activation phenotype [28].

Macrophages, as key regulators of cardiac inflammation, repair, and fibrosis within the cardiac microenvironment [29, 30, 31], accumulate in injured hearts via CCL2‐mediated CCR2^+^ monocyte recruitment [8, 32, 33]. Our scRNA‐seq results indicate that significant increases in CCR2^+^ macrophages after radiotherapy, immunotherapy, or combined treatment, which is consistent with observations in ICI‐related myocarditis, and targeting these cells significantly reduced pathogenic macrophage infiltration, and improved survival rates in mice [23]. Notably, we found infiltrated macrophages subjected to the combined therapy exhibit increased expression of CD86, while T lymphocytes showed an increase in CD28 expression level. This suggests macrophages as crucial mediators in the combined intervention induced cardiac injury, potentially enhancing interaction with autoreactive T cells, thereby modulating the injury process. Additionally, we observed a significant expansion of the Mmp12^+^ macrophages subtype following the combined intervention, which suggests the subset is associated with pro‐fibrotic processes in our scRNA‐seq. The role of MMP12 in fibrosis, however, remains controversial. While it is widely acknowledged for its anti‐inflammatory properties [34], and some studies link it to ECM degradation and fibrosis resolution [35], others have reported that MMP12 deficiency reduces fibrosis but paradoxically worsens cardiac function and survival [36, 37, 38]. This paradox highlights the complex, context‐dependent functions of MMP12 in cardiac remodeling.

Notably, our investigation into the cardiac microenvironment revealed that increased immune cells under the combined intervention can trigger local inflammation response and promote fibroblast activation. Specifically, under the chemotaxis of fibroblasts, immune cells, especially macrophages and T cells, are found to accumulate in cardiac tissues, and in turn regulate the activity of fibroblasts by secreting profibrotic factors, such as TGF‐β, PDGF, EGF, TNF, etc, which can contribute to the aggravation of cardiac fibrosis. Previous studies support the necessity of immune‐mediated fibroblast activation. In ischemia‐reperfusion (I/R) mouse models, CCR2^+^ macrophages from post‐I/R hearts upregulated Acta2/αSMA and Col1a2 in co‐cultured cardiac fibroblasts [39, 40]. Similarly, in idiopathic pulmonary fibrosis (IPF), alveolar fibroblast activation depends on adjacent macrophages, and direct co‐culture induces pro‐fibrotic signaling and fibroblast transformation [41]. Depletion of macrophages with clodronate liposomes, or inhibition of monocyte recruitment and macrophages development via CCR2 deficient mice reduced renal and myocardial fibrosis [42, 43, 44, 45]. Similarly, CD4^+^ T cells can also adhere to cardiac fibroblasts, and promote TGF‐β and IFN‐γ‐mediated myofibroblast transformation in heart failure models [46]. Together, these immune cells constitute central players in fibrotic pathogenesis. Elucidating the crosstalk between fibroblasts and immune cells under combined radioimmunotherapy is essential for understanding cardiac fibrosis and developing strategies to mitigate its complications.

Previous literatures have shown that IL‐6 mRNA is expressed at low baseline levels in cardiac fibroblasts but is undetectable in cardiomyocytes or macrophages. Its elevation is associated with hypertension, myocardial fibrosis, hypertrophy, heart failure, and diastolic dysfunction [47]. Interestingly, our scRNA‐seq data identified a distinct expression pattern of IL‐6 that was specifically enriched in fibroblasts and showed a progressive increase across the Con, ICI, IR, and iRT groups. IL‐6^+^ fibroblasts exhibit enhanced leukocyte differentiation, cell adhesion, cytokine/chemokine activities, TNF and NF‐kappa B signaling pathways, potentially promoting immune cell chemotaxis via the CXCL12/CXCL14_CXCR4 axis. Notably, these IL‐6^+^ fibroblasts engage in IL‐6 signaling pathway primarily interactions with themselves and macrophages, suggesting that while fibroblasts are activated by pro‐fibrotic factors secreted by macrophages, they in turn can activate macrophages via IL‐6 secretion. Previous co‐culture experiments demonstrated that macrophages induce IL‐6 production together with α‐SMA and collagen I expression in cardiac fibroblasts. This effect was attenuated by an IL‐6 neutralizing antibody, which reduced TGF‐β1 expression and Smad3 phosphorylation [47]. And IL‐6^−^/^−^ mice show reduced expression of α‐SMA, TGF‐β1, and collagen I in the heart, which reduces Ang II‐induced cardiac fibrosis [47]. Interestingly, our scRNA‐seq analysis of mice treated with the radioimmunotherapy combined with tocilizumab revealed that tocilizumab administration effectively blunted the activating effects of IL‐6^+^ fibroblasts on both themselves and CCR2^+^ macrophages.

Our scRNA‐seq was performed on cardiac samples from non‐tumor‐bearing mouse models, which may not fully represent the tumor microenvironment or tumor‐mediated systemic immune regulation. This choice was made to ensure experimental consistency across three endpoints and to overcome the technical challenges of long‐term observation in tumor‐bearing animals. Nevertheless, our supplementary experiments in LLC tumor‐bearing mice confirmed that tocilizumab combined with radioimmunotherapy not only alleviated cardiac injury and fibrosis but also enhanced antitumor efficacy, supporting the translational relevance of our findings. Additionally, while scRNA‐seq offers an extensive profile of non‐cardiomyocytes in myocardial tissue, its cardiomyocyte capture efficiency is constrained by their larger cell size. In the future, we need leverage single‐nucleus transcriptomics, or flow cytometry‐based sorting of cardiomyocytes from myocardial tissue to further investigate the specific impacts of radiotherapy combined with immunotherapy on myocardial injury. Additionally, the global IL‐6 knockout mice rather than fibroblast‐specific conditional knockout models were utilized in our study. Our preliminary data indicate that fibroblast‐derived IL‐6 accounts for the majority of total cardiac IL‐6 production (88.33%, Figure S10A and Table S5) in the iRT group, suggesting that global IL‐6 deficiency largely reflects the fibroblast contribution. Nevertheless, we acknowledge that the absence of a fibroblast‐specific IL‐6 knockout remains a limitation and that future studies employing conditional strategies will be necessary to further validate and extend our findings.

## 4 Conclusions

Our study elucidates the pivotal role of fibroblast–immune cell interactions, specifically highlighting IL‐6‐mediated crosstalk between cardiac fibroblasts and macrophages, as a key mechanism underlying radioimmunotherapy‐induced cardiac fibrosis. Importantly, tocilizumab demonstrated protective effects in preclinical models, underscoring its therapeutic potential. Further validation using fibroblast‐specific IL‐6 KO models and prospective clinical studies is warranted to optimize combination strategies and effectively manage cardiac adverse events.

## 5 Materials and Methods

### 5.1 Patients

This study recruited patients with malignant thoracic tumors who received thoracic radiotherapy at the Department of Oncology, the Second Affiliated Hospital of Nanchang University from October 2023 to January 2024. All patients were treated with intensity‐modulated radiation therapy (IMRT) or volumetric modulated arc therapy (VMAT) using the Elekta Versa HD linear accelerator (Stockholm, Sweden). Peripheral blood samples were systematically collected during two critical time points: within one week prior to radiotherapy initiation (baseline) and within 48 h following the final radiotherapy session. All samples underwent standardized processing through centrifugation at 3000 rpm for 15 min, with subsequent serum separation and aliquoting for storage at ‐80°C until analysis. The serum samples were subsequently quantitatively analyzed for the expression level of interleukin – 6 (IL‐ 6) using the enzyme‐linked immunosorbent assay (ELISA) method.

The final cohort enrolled 29 patients who met these inclusion criteria: (1) histopathological confirmation of thoracic malignancies, including lung cancer, esophagus cancer, breast cancer, and thymoma (2) completion of the full course of thoracic radiotherapy without interruption, and (3) availability of complete paired serum samples from both baseline and post‐radiotherapy time points. Comprehensive clinical data were collected, including demographic characteristics (gender, age), primary tumor pathology, cancer treatment history (incorporating chemotherapy, immunotherapy, and prior surgical interventions), and comorbidities (such as cardiovascular diseases and diabetes mellitus). The study protocol received formal approval from the Medical Research Ethics Committee of the Second Affiliated Hospital of Nanchang University (approval number: [2024]‐157), and written informed consent was obtained from all participants prior to their enrollment in the study.

### 5.2 Animal

Male wild‐type (WT) and IL‐6 knockout (KO) mice on a C57BL/6 background (GemPharmatech, Jangsu, China) aged 6–8 weeks, weighing 18–22 grams, were raised in the standardized environment of the Experimental Animal Centre of Nanchang University, with 5–6 mice per cage. The animal room was equipped with air conditioning to maintain a temperature of 23–24°C and moderate humidity. All cages were padded with corn kernels or wood shavings, and the bedding was changed on average once a week. Sufficient water and standard diet were provided daily.

Male wild‐type C57BL/6 mice (6‐8 weeks old) were randomly assigned to four experimental groups (*n* = 18 per group): (1) irradiation alone (IR), (2) ICI alone (ICI), (3) immunotherapy combined with irradiation (iRT), and (4) untreated control groups (Con). Mice in the IR and iRT groups underwent cardiac irradiation (CIR) using a small animal radiation research platform (SARRP; XStrahl, Gulmay Medical Systems, Suwanee, GA, USA). For longitudinal assessment of cardiac injury, functional evaluations were performed at three critical timepoints: acute phase (day 28), subacute phase (3 months), and chronic phase (5 months) post‐intervention, with 6 mice per group analyzed at each timepoint. Animal weights and clinical signs were monitored twice weekly throughout the treatment period to assess tolerability. Animals reaching experimental endpoints, including day 28, 3 months, and 5 months post‐intervention, tumor volume > 2000 mm^3^, or > 20% loss of initial body weight were humanely euthanized via cervical dislocation following deep anesthesia induced by intraperitoneal administration of 75 mg/kg pentobarbital sodium.

Additionally, to determine whether combined radioimmunotherapy induces cardiac injury and fibrosis in female mice, we extended our investigation to wild‐type C57BL/6J female mice subjected to the same experimental protocol. Furtherly, to investigate IL‐6‐dependent mechanisms, the same radiation and immunotherapy protocol was applied to IL‐6 KO mice, and pharmacological IL‐6 blockade was achieved in wild‐type mice through concurrent administration of tocilizumab during combined radioimmunotherapy (Toci). To investigate whether the mechanism of radioimmunotherapy‐induced cardiac fibrosis remains operative in the presence of tumors, and to examine the effect of tocilizumab on antitumor outcomes, we evaluated cardiac fibrosis and antitumor efficacy in mice bearing subcutaneous LLC tumors treated with tocilizumab in combination with radioimmunotherapy. All experimental procedures have received ethical approval from the Experimental Animal Committee of Nanchang University (NCULAE‐20221031142). All procedures were conducted in strict compliance with institutional animal care guidelines and reported according to ARRIVE Guidelines 2.0 (Supplementary material).

### 5.3 CIR Procedure

Prior to irradiation, mice were anesthetized by intraperitoneal injection of 75 mg/kg pentobarbital sodium and positioned in a supine orientation on the SARRP platform. High‐resolution computed tomography (CT) scans were acquired at 50 kV, 0.8 mA, with a 1.0 mm aluminum filter for precise anatomical localization. The heart, lungs, spinal cord, and other tissues were outlined on the CT images, and after evaluating that the dose for each tissue met the requirements, ARC field irradiation of 16 Gy was applied (equivalent to a square field of 10 mm × 10 mm, 220 kV, 13 mA, SSD = 345 mm, 0.15 mm copper filter). Post‐irradiation, mice were monitored for recovery under controlled conditions with supplemental warmth. The selected radiation dose was based on prior optimization studies and our previous work demonstrating marked induction of cardiac fibrosis while preserving survival [48]. To control for procedural effects, mice in the ICI and Con groups underwent identical anesthesia and positioning protocols but received 0 Gy sham irradiation. All irradiation procedures were performed by trained personnel following standardized protocols to ensure reproducibility.

### 5.4 In Vivo Treatments

To maintain stable circulating drug concentration, mice in the ICI and iRT groups received intraperitoneal injections of 200 µg anti‐mouse PD‐1 monoclonal antibody (BioXCell, clone RMP1‐14, West Lebanon, NH) every 3 days for 4 weeks (total 9 administrations per mouse). Control groups (IR and Con) were administered equivalent volumes of isotype‐matched IgG (BioXCell, clone 2A3, West Lebanon, NH) on the same schedule to control procedural consistency. The administration dosage of 200 µg anti‐PD‐1 monoclonal antibody was determined based on our previously established murine model of PD‐1 inhibitor‐induced myocardial injury [49, 50], and the selected drug frequency was to maintain stable blood concentration in mice [51]. The experimental protocol was also applied to both IL‐6 KO mice and WT C57BL/6J female mice to evaluate sex and IL‐6‐dependent responses. Additionally, to suppress the IL‐6/IL‐6R axis‐induced effects, mice were intraperitoneally injected with 10 mg/kg tocilizumab (Roche, Switzerland) every 3 days for 4 weeks in the tocilizumab (Toci) group, while receiving PD‐1 inhibitor with CIR intervention [12, 52, 53, 54].

### 5.5 Cardiac Echocardiographic Evaluation

Cardiac function was serially evaluated using high‐frequency ultrasound imaging at baseline and at day 28, 3 months, and 5 months post‐intervention. Prior to imaging, the region of thoracic and abdomen was carefully depilated to ensure optimal acoustic coupling. Mice were anesthetized using a standardized isoflurane protocol (3%–4% for induction, maintained at 1.5%–2%) and positioned in supine position on a temperature‐controlled platform within the Vevo 2100 ultrasound imaging system (VisualSonics, Canada). Ultrasound transmission gel was applied to the precordium, and a 40 MHz linear array transducer was used to obtain parasternal short‐axis views. M‐mode tracings were acquired at the papillary muscle level to measure the left ventricular diameter in diastole (LVID; d) and systole (LVID; s), left ventricle volume in diastole (LVVol; d) and systole (LVVol; s), interventricular septum in diastole (IVS; d) and systole (IVS; s), and the system automatically calculated the LVEF and LVFS. For echocardiographic assessment, three independent cardiac cycle images were captured for each mouse, with each image analyzed in triplicate to obtain averaged measurements, ensuring the reliability of the measurements.

### 5.6 Specimen Collection and Histological Analysis

The mice were sacrificed and cardiac tissues were collected for subsequent histological analysis at day 28, 3 months, and 5 months after the radioimmunotherapy intervention. The mice were anesthetized with an intraperitoneal injection of 75 mg/kg pentobarbital sodium, followed by cervical dislocation. The heart was removed, rinsed, followed by fixation in 4% paraformaldehyde, dehydration, embedding in paraffin, and preparation of paraffin sections. The paraffin blocks were sectioned and subjected to the histological analysis. Cardiac morphology and myocardial fibrosis were evaluated in H&E and Masson's trichrome staining, respectively. The infiltration of inflammatory cells was evaluated by immunohistochemical (IHC) inflammatory cell staining, including leukocytes (CD45^+^), macrophages (F4/80^+^), and T lymphocytes (CD8^+^ T and CD4^+^ T) staining. Sections were incubated with anti‐CD4 antibody (86300‐3‐RR, dilution 1:500, Proteintech, Wuhan, China), anti‐CD8 antibody (bsm‐60734R, dilution 1:500, Biosynthesis, Beijing, China), anti‐CD45 antibody (20103‐1‐AP, dilution 1:2000, Proteintech), and anti‐F4/80 antibody (dilution 1:100, ab111101, Abcam, Cambridge, UK). Pathological manifestations of cardiac injury were evaluated and confirmed by two independent, blinded pathologists. Quantitative analysis of IHC results was performed using FijiImage analysis software (National Institutes of Health, Bethesda, MD). At least three mice per group, and five representative fields per sample were used for histological assessment of cardiac tissues, and the images in three randomly microscopic (magnification, 400× and 200×) fields were analyzed and averaged.

### 5.7 Immunofluorescence Staining

Immunofluorescence staining was performed to evaluate the expression and spatial distribution of key fibrotic and inflammatory markers in cardiac tissue and cellular sections. Primary antibodies targeting anti‐TGF‐β1 (81746‐2‐RR, dilution 1:500, Proteintech), anti‐α‐SMA (80008‐1‐RR, dilution 1:500, Proteintech), anti‐Fn1 (bs‐0666R, dilution 1:500, Biosynthesis,), anti‐Collagen I (ab270993, dilution 1:2000, Abcam), anti‐Collagen III (ab184993, dilution 1:100, Abcam), anti‐F4/80 (ab111101, dilution 1:500, Abcam), anti‐CD3 (ab16669, dilution 1:100, Abcam), and anti‐IL‐6 (ab290735, dilution 1:50, Abcam), and CD86 (bs‐43589R, dilution 1:200, Biosynthesis) were applied at optimized dilutions. Following thorough washing, appropriate species‐matched secondary antibodies were applied for signal amplification. The staining score was calculated as the overall value of the staining intensity by 2 independent and experienced pathologists. Per sample were analyzed at 400× magnification to balance the tissue heterogeneity. The integrated positive area within the region of interest was analyzed to assess the staining intensity using FijiImage analysis software (National Institutes of Health, Bethesda, MD). The colocalization score was analyzed via the colocalization plugin of FijiImageJ software.

### 5.8 Enzyme‐Linked Immunosorbent Assay (ELISA)

Cardiac tissue and serum samples were collected from murine models undergoing radioimmunotherapy intervention for cardiac function and IL‐6 expression analysis. Mouse whole blood samples were allowed to clot overnight at 4°C, followed by centrifugation at 1500 × g for 15 min to isolate serum, which was aliquoted and stored at −80°C until analysis. Human serum samples from thoracic malignancy patients undergoing radiotherapy were processed identically. Additionally, for mouse heart tissue in the different intervention groups, an equal amount of 30 mg of left ventricular myocardial tissue was precisely measured and weighed. The tissue was then submerged in an extraction buffer, containing protease inhibitors, at a ratio of 10:1 (buffer volume to tissue weight). The mixture underwent homogenization and lysis on ice. Subsequently, the homogenized tissue samples were centrifuged at 5000 g for 15 min at 4°C to separate and discard tissue debris and impurities.

Serum cTnI and NT‐proBNP levels in mice were quantified using commercial ELISA kits (Elabscience, Wuhan, China), while IL‐6 concentrations in both murine cardiac lysates and human serum were measured using specific ELISA kits (Elabscience, and Abcam, respectively). Standard curves were generated for each assay, and sample concentrations were calculated using four‐parameter logistic regression. All experimental procedures included appropriate controls and were performed with three technical replicates to ensure data reliability.

### 5.9 scRNA‐seq and Buk RNA‐seq

The study employed both single‐cell and bulk RNA sequencing approaches to comprehensively characterize cardiac tissue transcriptomes. Cardiac tissues were harvested at the day 28 post‐intervention timepoint across control and experimental groups. For scRNA‐seq, myocardial tissues from five experimental groups (Con, ICI, IR, iRT, and Toci) were processed, with three biological replicates per group yielding a total of 15 specimens. For bulk transcriptome sequencing, cardiac tissues from three experimental groups (Con, iRT, and Toci) were analyzed with three biological replicates per group using the Illumina NovaSeq 6000 platform.

To prepare the cardiac tissues for scRNA‐seq, tissues were dissociated into the high‐quality single‐cell suspensions using a standardized enzymatic and mechanical protocol. The scRNA‐seq libraries were prepared following manufacturer protocols by GEXSCOPE Single Cell RNA Library Kit (Singleron Biotechnologies) and performed using the Illumina NovaSeq 6000 (150 bp paired‐end reads). Initial data processing involved quality filtering, followed by alignment to the 10‐mm reference genome using CeleScepe v1.5.2 (Singleron Biotechnologies) with default parameters. The analysis of single‐cell datasets begins with the application of mechanical clustering, dimensionality reduction for visualization, and cell annotation to identify cell types and their secondary subtypes within cardiac tissues. Based on the characteristics of single‐cell expression profiles and the expression of cell marker genes, we employ an automated annotation approach coupled with manual review and calibration to assign identity labels to each cell. The detailed protocols for tissue processing, sequencing methodologies, and data analysis methods, including detailed quality control metrics, integration parameters, clustering resolutions, and downstream bioinformatic approaches, are comprehensively documented in the Supplementary Materials to ensure methodological reproducibility.

### 5.10 Gene Sets for ECM and Pro‐Fibrotic Molecules

Integrated gene sets encompassing ECM components and pro‐fibrotic mediators were curated to systematically quantify ECM remodeling and fibrotic activation signatures across cardiac single‐cell populations and bulk RNA‐seq data. The ECM gene set comprised both structural and regulatory molecules, systematically curated through integration of published literature [19] and Molecular Signatures Database (MSigDB, NABA_COLLAGENS, MM17060), including collagens (*Col1a1, Col1a2, Col3a1, Col4a1–Col4a6, Col5a1–Col5a3, Col6a1–Col6a3, Col6a5, Col6a6, Col7a1, Col8a1, Col8a2, Col9a1‐Col9a3, Col11a1, Col11a2, Col12a1, Col13a1, Col14a1, Col15a1, Col16a1, Col17a1, Col18a1, Col19a1, Col20a1, Col22a1, Col23a1, Col24a1, Col25a1, Col26a1, Col27a1*, *Col28a1*), laminins (*Lama2–Lama5, Lamb1–Lamb3, Lamc1–Lamc3*), and glycoproteins (*Fn1, Cthrc1, Mfap2, Ntn1, Tnc, Postn, Lum*). The pro‐fibrotic gene set was compiled from published literature [14, 15, 16, 17, 18, 19] and categorized into four functional classes: (1) cytokines (*Tnf, Ifng, Lif, Il1a, Il1b, Il4, Il5, Il6, Il9, Il11, Il13, Il17a, Il25, Il33, Osm*); (2) chemokines (*Cxcl1, Cxcl2, Cxcl5, Cxcl9–Cxcl13, Ccl2, Ccl5, Ccl8, Ccl18, Ccl19, Ccl21a*); (3) growth factors (*Tgfb1, Pdgfa, Fgf2, Ctgf, Egf, Igf1, Serpine1, Acta2, Vegfa, Lgals3, AngII, Wnt, Ras*); and (4) matrix metalloproteinases (*Mmp2, Mmp3, Mmp7, Mmp9, Mmp11‐Mmp14, Mmp19*).

### 5.11 In Vivo Tumor Imaging in Mice Bearing LLC Tumors

Six‐week‐old male C57BL/6J mice (body weight 16–20 g) were anesthetized with 2% isoflurane and subcutaneously inoculated with 0.15 mL of luciferase‐expressing LLC (#IML‐036, Immocell, Fujian, China) suspension (5 × 10^6^ cells/mL in sterile PBS). The LLC cell line was authenticated via short tandem repeat (STR) profiling and confirmed to be free of mycoplasma and chlamydia contamination. The cells were inoculated at 2 cm below the right posterior axillary line. Tumor growth was monitored daily using caliper measurements. When tumors reached ∼100 mm^3^, mice were randomly allocated into experimental groups or Control group (*n* = 6–8/group). Tumor bioluminescence signals were measured using the IVIS Lumina Series III system (PerkinElmer, Waltham, USA) at the beginning of the administration and 4 weeks after the administration to assess tumor size. For imaging sessions, animals were anesthetized with 2% isoflurane and intraperitoneally administered D‐luciferin (150 mg/kg; #MB1834, MeilunBio, Dalian, China) 10–15 min prior to image acquisition. Photon flux was quantified using Living Image software (version 4.4, PerkinElmer) with consistent exposure parameters (30 s acquisition, medium binning).

### 5.12 Cell Culture and Model Construction

RAW 264.7 murine macrophages (#CL‐0190, Procell Life Science & Technology) were authenticated by STR profiling and confirmed to be free of mycoplasma and chlamydia contamination prior to use. Cells were cultured in high‐glucose Dulbecco's Modified Eagle Medium (DMEM, Gibco, USA) supplemented with 10% fetal bovine serum (FBS, Gibco, USA) and 1% penicillin‐streptomycin (Gibco, USA) at 37°C under a humidified atmosphere containing 5% CO_2_. Cells were subcultured at 90% confluence by washing with PBS, mechanically detached using a cell scraper, and centrifuged at 1000 rpm for 5 min. After supernatant removal, cells were resuspended in fresh complete medium and passaged at a 1:3 ratio into new T25 flasks containing 5 mL medium. For experimental treatments, logarithmically growing cells were seeded in 6‐well plates at a density of 5 × 10^5^ cells per well with triplicate wells for each condition. After 12‐h stabilization, the culture medium was replaced with fresh medium containing recombinant murine IL‐6 (#216‐16‐10, PeproTech, Rocky Hill, NJ, USA) at concentrations of 0, 20, 30, 50, and 100 ng/mL, with a final volume of 2 mL per well. After 48 h of treatment, cells were harvested for subsequent fluorescence staining analysis.

Primary cardiac fibroblasts (#CP‐M074, Procell Life Science & Technology) were isolated from the hearts of 1‐3‐day‐old C57BL/6 mice and authenticated via immunofluorescence staining, which confirmed positive expression of vimentin. Cells were cultured in fibroblast‐specific complete medium (#CM‐M074, Procell) at 37°C with 5% CO_2_. At approximately 90% confluence, cells were trypsinized using 0.25% trypsin‐EDTA (Gibco, USA) and subcultured in 6‐well plates or T25 flasks for subsequent experiments. According to experimental design, cells were treated with fresh medium containing different concentrations (0 or 100 ng/mL [55, 56]) of recombinant murine IL‐6 (#216‐16‐10, PeproTech). To assess the JAK2/STAT3 pathway activation, fibroblasts were incubated with 1 µm Ruxolitinib (#S1378, Selleck, Houston, Texas, USA) for 1 h, followed by co‐incubation with 100 ng/mL IL‐6 for 48 h [57, 58]. After 48 h of treatment, cells were harvested for fibrosis‐related molecular assays.

### 5.13 Quantitative Reverse Transcription‐Polymerase Chain Reaction (qRT‐PCR) Analysis

Total RNA was extracted from cardiac tissue specimens utilizing TransZol Up Plus RNA Kit (TransGen Biotech, Beijing, China) according to the manufacturer's protocol. Following quality assessment, 2 µg of high‐quality total RNA from each sample was reverse transcribed into complementary DNA (cDNA) using a commercial reverse transcription kit (TransGen Biotech). Quantitative PCR amplification was subsequently performed in triplicate using SYBR Premix EX Taq II (Takara Biomedicals, Shiga, Japan) on a CFX96 Real‐Time PCR Detection System (Bio‐Rad Laboratories, Hercules, CA, USA). Target gene expression levels were quantified via the comparative Ct (ΔΔCt) method, with β‐Actin serving as the endogenous reference gene for normalization. Three independent experimental replicates were conducted to ensure reproducibility. The primer sequences employed for amplification were as follows: IL‐6 (forward: 5′‐CTGCAAGAGACTTCCATCCAG‐3′; reverse: 5′‐ AGTGGTATAGACAGGTCTGTTGG‐3′), col1a1 (forward: 5′‐GAGAGGTGAACAAGGTCCCG‐3′; reverse: 5′‐AAACCTCTCTCGCCTCTTGC‐3′), Col3a1 (forward: 5′‐GTGGCAATGTAAAGAAGTCTCTGAAG‐3′; reverse: 5′‐GGGTGCGATATCTATGATGGGTAG‐3′), TGF‐β1 (forward: 5′‐ACTGGAGTTGTACGGCAGTG‐3′; reverse: 5′‐GGCTGATCCCGTTGATTTCC‐3′), α‐SMA (forward: 5′‐GTCCCAGACATCAGGGAGTAA‐3′; reverse: 5′‐TCGGATACTTCAGCGTCAGGA‐3′), and β‐Actin (forward: 5′‐GTGACGTTGACATCCGTAAAGA‐3′; reverse: 5′‐GTAACAGTCCGCCTAGAAGCAC‐3′).

### 5.14 Western Blot

Cellular and cardiac tissue samples were homogenized in a 1:1:100 configuration of protease inhibitor: phosphatase inhibitor: radio‐immunoprecipitation assay (RIPA) lysis buffer. Following complete lysis, cellular debris was removed by centrifugation at 12,000 × g for 15 min at 4°C, and the resulting supernatant was collected for downstream analysis. Protein concentrations were determined using the bicinchoninic acid (BCA) assay, with samples subsequently normalized to equal concentrations for comparative analysis. Normalized protein samples were resolved by SDS‐PAGE (8%–15% gradient gels) under denaturing conditions. Separated proteins were then electrophoretically transferred to PVDF membranes using a wet transfer system. Non‐specific binding sites were blocked by incubating membranes with 5% skim milk or 5% BSA in Tris‐buffered saline containing 0.1% Tween‐20 (TBST) for 60 min at room temperature with gentle agitation. PVDF membranes were incubated overnight at 4°C with primary antibodies, washed with TBST, then probed with species‐matched horseradish peroxidase (HRP)‐conjugated secondary antibodies for 1 h at room temperature. Protein bands were visualized using a Bio‐Rad gel imaging system. Band intensity quantification was performed using ImageJ software (National Institutes of Health, Bethesda, MD).

The following primary antibodies were used: anti‐Collagen I (ab270993, dilution 1:2000, Abcam,), anti‐Collagen III (ab184993, dilution 1:1000, Abcam), anti‐TGF‐β1 (81746‐2‐RR, dilution 1:2000, Proteintech, and ab315254, dilution 1:1000, Abcam), anti‐α‐SMA (80008‐1‐RR, dilution 1:20000, Proteintech), anti‐IL‐6 (ab290735, dilution 1:1000, Abcam), anti‐β‐actin (66009‐1‐Ig, dilution 1:30000, Proteintech), anti‐JAK2 (ab108596, dilution 1:1000, Abcam), anti‐STAT3 (ab68153, dilution 1:1000, Abcam), anti‐p‐JAK2 (ab32101, dilution 1:500, Abcam), anti‐p‐STAT3 (ab267373, dilution 1:500, Abcam). The secondary antibodies used were anti‐rabbit (SA00001‐2, dilution 1:5000, Proteintech) and anti‐mouse IgG (SA00001‐1, dilution 1:10000, Proteintech).

### 5.15 Statistical Analysis

Categorical variables were expressed as counts and percentages (%), and continuous variables were presented as mean ± standard deviation (SD) or median (interquartile range [IQR]). For non‐normally distributed numerical variables, paired‐sample comparisons were performed using the Wilcoxon signed‐rank test. Normally distributed variables were compared between two groups using Student's t‐test (independent samples). For scenarios involving multiple groups, one‐way analysis of variance (ANOVA) accompanied by pairwise comparisons was carried out. The overall survival was evaluated by the Kaplan‒Meier method. All statistical tests were two‐sided, and a *p* value < 0.05 was considered statistically significant. Statistical analysis was performed utilizing R software (version 4.3.1) and GraphPad Prism (version 8.3).

## Author Contributions

Y.L., L.C. and A.L. contributed to the conceptualization, study design, and work execution. Y.L. performed the experiment, conducted data analysis, and drafted the original draft. L.C. and A.L. contributed to the funding acquisition, writing – review and editing, and discussion. Y.L., Z.Z., Y.C., and P.X. collected clinical information and blood samples, and Y.Y., Y.Y., Z.L., and F.Z. assisted with animal experiments. D.L. and Z.Z. contributed to the data interpretation and supervision. All authors read and approved the final version of the manuscript.

## Conflicts of Interest

The authors declare no conflicts of interest.

## Supporting information




**Supporting File**: advs74497‐sup‐0001‐SuppMat.docx.

**Supporting File**: advs74497‐sup‐0002‐Tables.xlsx.

## Data Availability

The data that support the findings of this study are available from the corresponding author upon reasonable request.
