# Supplementary material for: Decoding the Cardiac Immune Microenvironment and Fibroblast Crosstalk in Radiotherapy Combined with Immunotherapy‐Induced Cardiac Fibrosis Based on Single‐Cell Transcriptomic Analysis
Source: Adv Sci (Weinh). 2026 Feb 20;13(25):e19216. doi: 10.1002/advs.202519216 (PMC13137807; doi:10.1002/advs.202519216)
Supplement: Supplementary file 1 — Supporting File: advs74497‐sup‐0001‐SuppMat.docx. [file ADVS-13-e19216-s002.docx]

**Supplementary Materials for**

**Decoding the cardiac immune microenvironment and fibroblast crosstalk in radiotherapy combined with immunotherapy-induced cardiac fibrosis based on single-cell transcriptomic analysis**

**Materials and Methods**

**Tissue Dissociation and Preparation of scRNA-seq**

The fresh tissues of mouse heart were immediately stored in the sCelLiveTM Tissue Preservation Solution (Singleron) within 5 minutes of removal. The specimens underwent a triple rinsing process with Hanks Balanced Salt Solution (HBSS), followed by meticulous dicing into fine fragments, and then digested with 3 mL sCelLiveTM Tissue Dissociation Solution (Singleron) by Singleron PythoN™ Tissue Dissociation System at 37 °C for 15 min. The cell suspension was collected and filtered through a 40-micron sterile strainer. Subsequently, the GEXSCOPE® red blood cell lysis buﬀer (RCLB, Singleron) was added into the cell suspension at a 1:2 volume ratio (Cell:RCLB). The mixture was then incubated at room temperature for 5- 8 minutes to effectively eliminate red blood cells. The mixture was then centrifuged at 300 × g 4 ℃ for 5 mins to remove supernatant and suspended softly with PBS. Finally, the samples were stained with Trypan Blue, the cell viability was evaluated microscopically, and once it exceeds 80%, subsequent sample processing can be carried out.

**The Construction of scRNA-seq Library and Primary Analysis of Raw Read Data**

The single-cell suspensions (1×10^5^ cells/mL) with PBS were loaded onto a microfluidic device using the Singleron Matrix^®^ Single Cell Processing System. Then, the scRNA-seq library was constructed according to the Singleron GEXSCOPE^®^ protocol through the GEXSCOPE^®^ Single-Cell RNA Library Kits (Singleron Biotechnologies) [1], including cell lysis, mRNA capture, tagging cells (barcodes), and mRNA (UMI), reverse transcription of mRNA into cDNA and amplification. The amplified cDNA is then fragmented and ligated with sequencing adapters. The individual libraries were diluted to 4 nM, pooled, and sequenced on Illumina novaseq 6000 with 150 bp paired end reads.

Raw reads from scRNA-seq were processed to generate gene expression matrixes utilizing CeleScope v1.5.2 from Singleron Biotechnologies (https://github.com/singleron-RD/CeleScope) with default parameters. Initially, raw reads were first processed with CeleScope to remove low quality reads with Cutadapt v1.17 [2] to trim poly-A tail and adapter sequences. Cell barcode and UMI were extracted.

The refined reads were then mapped to the GRCm38 reference genome (ensembl version 92 annotation) employing STAR v2.6.1a [3]. UMI counts and gene counts per cell were acquired with featureCounts v2.0.1 software [3], and used to generate expression matrix files for subsequent analysis.

**Quality Control, Dimension-reduction and Clustering (Scanpy)**

Quality control, dimensionality reduction, and clustering were performed using Scanpy [4] v1.8.2 under Python 3.9. For each sample dataset, the expression matrix was filtered according to the following criteria: 1) cells with gene count less than 200 or with top 2% gene count were excluded; 2) cells with top 2% UMI count were excluded; 3) cells with mitochondrial content greater than 30% were excluded; 4) genes expressed in less than 5 cells were excluded. After filtering, 111566 cells were retained for the downstream analyses, with an average 1205 genes and 2716 UMIs per cell. The raw count matrix was normalized based on the total counts per cell and logarithmically transformed into a normalized data matrix. The top 2000 variable genes were selected by setting flavor as ‘seurat’. Principle Component Analysis (PCA) was carried out on the scaled variable gene matrix, and top 26 principle components were employed for clustering and dimensional reduction. Cells were divided into 26 clusters by using Louvain algorithm with the resolution parameter set at 1.2. Cell clusters were visualized via Uniform Manifold Approximation and Projection (UMAP) {t-Distributed Stochastic Neighbor Embedding (t-SNE)}.

**Differentially Expressed Genes (DEGs) Analysis (scanpy)**

To identify DEGs, we applied the scanpy.tl.rank_genes_groups() function based on Wilcoxon rank sum test with default parameters, and selected the genes expressed in more than 10% of the cells in either of the compared groups of cells and with an average log(Fold Change) value＞1 as DEGs. Statistical significance was assessed using a Benjamini-Hochberg adjusted *P*-value threshold of 0.05. For the cell type annotation of each cluster, we integrated the expression profiles of canonical markers derived from the DEGs with existing literature knowledge. Subsequently, we visualized the expression patterns of these markers for each cell type with heatmaps/dot plots/violin plots that were generated with Seurat DoHeatmap/DotPlot/Vlnplot function. Doublet cells were identified as expressing markers for different cell types, and removed manually.

**Pathway Enrichment Analysis**

To delve into the potential functions of DEGs, Gene Ontology (GO) and Kyoto Encyclopedia of Genes and Genomes (KEGG) analysis were used with the “clusterProfiler” R package v 4.0.0 [5]. Pathways exhibited with an adjusted p-value (p_adj) below 0.05 were considered as significantly enriched. Subsequently, selected enriched pathways were visually represented as bar plots for clarity. Gene Ontology gene sets spanning molecular function (MF), biological process (BP), and cellular component (CC) categories were used as reference.

**Celltype Annotation**

**Cell-type Recognition with Cell-ID**

Cell-ID is multivariate approach that extracts gene signatures for single cell and employs hypergeometric tests (HGT) for cell identity recognition. Dimensionality reduction of the normalized gene expression matrix was carried out via multiple correspondence analysis, projecting both cells and genes in the shared low-dimensional space. Then, a gene ranking was calculated for each cell to identify its most featured gene sets. HGT were performed on these gene sets using mouse cardiac tissue reference from SynEcoSys database, which contains all cell-type's featured genes in the mouse cardiac tissue. The identity of each cell was determined as the cell-type with the minimal HGT p value. For cluster annotation, the frequency of each cell-type was calculated in each cluster, and the cell-type with the highest frequency was selected as the cluster's identity. The cell type identity of each cluster was determined with the expression of canonical markers from the reference database SynEcoSysTM (Singleron Biotechnology), which contains extensive collections of markers from CellMakerDB, PanglaoDB, and recent literature publications, tailored specifically for single-cell sequencing data [6-7].

**Subtyping of Major Cell Types**

To obtain a high-resolution map of cell subpopulation, cells from the specific cluster were extracted and reclustered for more detailed analysis following the same procedures described above, with the clustering resolution set as 1.2 [7].

**Filtering Cell Doublets and RNA Contamination**

To ensure data veracity, we estimated and mitigated the presence of cell doublets and RNA contamination. First, clusters exhibiting an expression pattern enriched with multiple cell type-specific canonical markers were excluded from further analysis. Secondly, to reduce the influence of RNA contamination and doublets in the downstream analysis, DecontX [8] was applied to estimate and remove contamination, while DoubletFinder [9] was utilized to identify and exclude doublets.

**Definition of IL-6^+^ Fibroblasts and CCR2^+^ Macrophages**

Following cell type annotation, we conducted subset-specific analyses. To investigate the role of IL-6-expressing fibroblasts, we stratified the cardiac fibroblast population into IL-6⁺ and IL-6⁻ subsets based on transcriptional activity. Fibroblasts with detectable expression of the *Il6* gene (normalized UMI count > 0) were classified as “IL-6⁺ fibroblasts”. Conversely, fibroblasts with no detectable Il6 transcript (normalized UMI count = 0) were defined as “IL-6⁻ fibroblasts”. This binary classification was used for all subsequent comparative analyses between these fibroblast subpopulations. Similarly, macrophages were stratified into CCR2⁺ and CCR2⁻ subsets based on the same principle. Cells with detectable *Ccr2* gene expression (normalized UMI count > 0) were designated as CCR2⁺ macrophages, whereas those without detectable expression (normalized UMI count = 0) were defined as CCR2⁻ macrophages.

**Cell-cell Interaction Analysi**s (**CellChat**)

CellChat (version 1.6.1) was employed to analyze the intercellular communication networks based on the receptor-ligand interaction between two cell types/subtypes from scRNA-seq data [10]. A CellChat object was constructed using the R package process. Cell information was incorporated into the meta slot of the object. The ligand-receptor interaction database was configured, and the matching receptor inference calculation was conducted.

**Cell-cell Interaction Analysis (CellPhoneDB)**

The cell-cell interaction analysis was carried out by CellPhoneDB v4.0.0 [11] on the basis of the known receptor–ligand interactions between two cell types/subtypes. The cluster labels of all cells were randomly permuted for 1000 times to calculate the null distribution of average ligand-receptor expression levels of the interacting clusters. Individual ligand or receptor expression was thresholded using a cutoff value based on the average log gene expression distribution for all genes across all cell types. The criterion for identifying significant cell-cell interactions was set at a *P* value < 0.05 and an average log expression > 0.1. These interactions were visualized by utilizing the circlize v0.4.10 R package for analysis.

**Expression Pattern Cluster**

We utilized Mfuzz v2.46.0 to decipher treatment-related transcriptional programs in various cells types. The samples were categorized based on different interventions. We commenced by calculating the average expression of each gene for each group, excluding genes with more than 25% missing values. Replace the remaining deletion values with the mean expression values of the corresponding genes. Then, the “filter.std(min.std=0)” and “standardize()” functions were performed for preprocessing according to the tutorial.

**UCell Gene Set Scoring**

Gene set scoring was performed using the R package UCell v 2.2.0 [12]. UCell scores are based on the Mann-Whitney U statistic by ranking query genes’ in order of their expression levels in individual cells. Because UCell is a rank-based scoring method, it is suitable to be used in large datasets containing multiple samples and batches. Analytically relevant gene sets were curated to include a pro-fibrotic gene set (excluding matrix metalloproteinases, MMPs) and an extracellular matrix (ECM) gene set.

**RNA Extraction and Library Construction of Bulk RNA-seq**

After the extraction of mouse heart tissue, it was promptly frozen in liquid nitrogen for 10-15 minutes and subsequently stored at -80°C to preserve for bulk transcriptome sequencing. Total RNA isolation and purification were performed using TRIzol reagent (Invitrogen, Carlsbad, CA, USA) according to the manufacturer's procedure. The quantity and purity of RNA in each sample were accurately determined with NanoDrop ND-1000 (NanoDrop, Wilmington, DE, USA). The RNA integrity was evaluated by Bioanalyzer 4200 (Agilent, CA, USA) with RIN number >6.0, and verified via electrophoresis on denaturing agarose gel. Poly (A) RNA was isolated from 0.1-4μg total RNA employing VAHTS mRNA Capture Beads 2.0 N403-02 (Vazyme, China) through double purification cycles. The poly(A) RNA was subsequently fragmented into small segments utilizing VAHTS Universal V8 RNA-seq Library Prep Kit for Illumina (Vazyme, China) under 94℃ 5min. Then the cleaved RNA fragments were reverse-transcribed to create the cDNA by VAHTS Universal V8 RNA-seq Library Prep Kit for Illumina (Vazyme, China), which were next used to synthesise U-labeled second-stranded DNAs with VAHTS Universal V8 RNA-seq Library Prep Kit for Illumina (Vazyme, China). An A-base was then appended to the blunt ends of each strand, preparing them for ligation to the indexed adapters. Each adapter contains a T-base overhang for ligating the adapter to the A-tailed fragmented DNA. Single- or dual-index adapters are ligated to the fragments, and size selection was performed with VAHTS DNA Clean beads. After the heat-labile UDG enzyme (NEB, cat.m0280, USA) treatment of the U-labeled second-stranded DNAs, the ligated products are amplified with PCR. The PCR protocol begins with an initial denaturation step at 98°C for 45 sec to separate the DNA strands; This is followed by 14 cycles of denaturation at 98°C for 15 sec, annealing at 60°C for 30 sec to allow primer binding, and extension at 72°C for 30 sec for DNA synthesis; Finally, a final extension step at 72°C for 1 minutes completes the PCR amplification. The final cDNA library exhibited an average insert size of 300±50 bp. At last, we conducted the 2×150bp paired-end sequencing (PE150) on an illumina Novaseq™ Xplus following the vendor's recommended protocol.

**Bioinformatics Analysis of Bulk RNA-seq**

For quality control of the raw data, the fastp software (https://github.com/OpenGene/fastp) were used to remove the reads that contained adaptor contamination, low quality bases and undetermined bases with default parameter. The sequencing data was subsequently aligned to the reference genome of *Mus musculus* GRCm38, via HISAT2 (https://ccb.jhu.edu/software/hisat2), and generating BAM files. Using the StringTie software (https://ccb.jhu.edu/software/stringtie), genes or transcripts were assembled and quantified with FPKM (FPKM = total_exon_fragments / mapped_reads(millions) × exon_length(kB)) . The differential gene expression analysis was performed between samples using the R package edgeR (https://bioconductor.org/packages/release/bioc/html/edgeR.html), with genes defined as differentially expressed if they exhibited the fold change > 2 or fold change < 0.5, and p-value < 0.05. Finally, the DAVID software (https://david.ncifcrf.gov/) was utilized to conduct GO and KEGG enrichment analyses on the identified genes. In parallel, Gene Set Variation Analysis (GSVA) was executed to assess pathway enrichment, leveraging the average gene expression profiles of each cell types as input data.

1. Dura B, Choi JY, Zhang K, Damsky W, Thakral D, Bosenberg M, Craft J, Fan R. scFTD-seq: freezethaw lysis based, portable approach toward highly distributed single-cell 3' mRNA profiling. Nucleic Acids Res. 2019 Feb 20;47(3):e16.

2. Martin M. (2011) Cutadapt removes adapter sequences from high-throughput sequencing reads. EMBnet J, 17.10-12.

3. Dobin A, Davis CA, Schlesinger F, Drenkow J, Zaleski C, Jha S, Batut P, Chaisson M, Gingeras TR. STAR: ultrafast universal RNA-seq aligner. Bioinformatics. 2013 Jan 1;29(1):15-21.

4. Wolf FA, Angerer P, Theis FJ. SCANPY: large-scale single-cell gene expression data analysis. Genome Biol. 2018 Feb 6;19(1):15. doi: 10.1186/s13059-017-1382-0.

5. Yu G, Wang LG, Han Y, He QY. clusterProfiler: an R package for comparing biological themes among gene clusters. OMICS. 2012 May;16(5):284-7.

6. Cortal A, Martignetti L, Six E, Rausell A. Gene signature extraction and cell identity recognition at the single-cell level with Cell-ID. Nat Biotechnol. 2021 Sep;39(9):1095-1102.

7. Yan Zhang, Bingyu Li, Jiachen Duan, Xuezhen Chen, Xiaogang Zhang, Jun Ye, Ana Veloso, Jue Fan, Nan Fang. Preprint at bioRxiv <https://doi.org/10.1101/2023.02.14.528566>.

8. Yang S, Corbett SE, Koga Y, Wang Z, Johnson WE, Yajima M, Campbell JD. Decontamination of ambient RNA in single-cell RNA-seq with DecontX. Genome Biol. 2020 Mar 5;21(1):57.

9. McGinnis CS, Murrow LM, Gartner ZJ. DoubletFinder: Doublet Detection in Single-Cell RNA Sequencing Data Using Artificial Nearest Neighbors. Cell Syst. 2019 Apr 24;8(4):329-337.e4.

10. Jin S, Guerrero-Juarez CF, Zhang L, Chang I, Ramos R, Kuan CH, Myung P, Plikus MV, Nie Q. Inference and analysis of cell-cell communication using CellChat. Nat Commun. 2021 Feb 17;12(1):1088.

11. Efremova M, Vento-Tormo M, Teichmann SA, Vento-Tormo R. CellPhoneDB: inferring cell-cell communication from combined expression of multi-subunit ligand-receptor complexes. Nat Protoc. 2020 Apr;15(4):1484-1506.

12. Andreatta, Massimo, and Santiago J. Carmona. "UCell: Robust and scalable single-cell gene signature scoring." Computational and Structural Biotechnology Journal 19 (2021): 3796-3798.

**ARRIVE Essential 10.**

| **ARRIVE Essential 10** | | |
| --- | --- | --- |
| Study design | 1 | Group allocation: A total of 72 male C57BL/6 mice (age 6-8 weeks) were randomly allocated into four experimental groups (n=18 per group) using a computer-generated randomization sequence to minimize bias. The groups consisted of: (1) Con (intraperitoneal administration of IgG isotype control antibody); (2) ICI (anti-PD-1 monoclonal antibody monotherapy, 200 µg every 3 days); (3) IR (focal cardiac irradiation alone, 16 Gy single fraction); and (4) iRT (combined anti-PD-1 therapy and cardiac irradiation).  Housing standardization: Animals were group-housed (6 mice per cage) in individually ventilated cages under specific pathogen-free (SPF) conditions, with controlled temperature (22±1°C), humidity (50±10%), and a 12-hour light/dark cycle. Each cage of mice was kept in the same house conditions, and each cage of mice was given the same autoclaved standard chow and sterile water.  Timing of the experiment: To assess longitudinal outcomes, a pre-defined euthanasia schedule was implemented with terminal timepoints at day 28 (acute phase), 3 months (intermediate), and 5 months (late effects) post-intervention (n=6 per group per timepoint). All procedures were approved by the Experimental Animal Committee of Nanchang University (NCULAE-20221031142) and complied with ARRIVE 2.0 and NIH guidelines. |
| Sample size | 2 | Experimental cohort structure: The study employed a total of 72 male C57BL/6J mice to ensure adequate statistical power across four experimental groups (n=18 per group). Each experimental group incorporated three temporal cohorts (n=6 per cohort) representing distinct pathophysiological phases: acute (day 28 post-intervention), subchronic (3 months), and chronic (5 months) responses. The n=6 per timepoint was maintained consistently across all groups to ensure balanced comparisons, which was a reference to our previous study (PMID: 34633055). |
| Inclusion and exclusion  criteria | 3 | Prior to randomization, all C57BL/6J mice underwent comprehensive pre-intervention screening to ensure cohort homogeneity. Exclusion criteria were rigorously applied to: (1) animals demonstrating baseline echocardiographic abnormalities (left ventricular ejection fraction [LVEF] <45% or fractional shortening [LVFS] <25% via transthoracic echocardiography under 1.5% isoflurane anesthesia), and (2) subjects falling outside the standardized weight parameters (18-22 g) during acclimatization. |
| Randomization | 4 | Randomization: A randomization protocol was implemented to ensure balanced group allocation across all experimental conditions. Baseline group allocation was performed using the RAND() function in Microsoft Excel 2021 to generate unbiased randomization sequences.  Confounder control measures: To minimize confounding variables, all experimental procedures including cardiac ultrasound assessments (performed under 1.5% isoflurane anesthesia), intraperitoneal PD-1 inhibitor administrations (200 µg), and cardiac irradiation protocols (16 Gy single fraction) were consistently conducted during the morning circadian phase (08:30-12:00) under standardized environmental conditions (22±1°C, 50±10% humidity). |
| Blinding | 5 | To minimize potential bias and ensure objective data collection, a triple-blinded experimental design was implemented throughout the study. Three independent investigators were sequentially involved in distinct phases of the research workflow to maintain strict separation between allocation, intervention, and assessment procedures.  Group allocation: The first investigator assigned animals to four groups strictly following a predefined randomization schedule.  Intervention implementation: The second investigator executed all intervention procedures, including intraperitoneal administration of either PD-1 inhibitor or IgG isotype control, as well as cardiac irradiation.  Blinded outcome assessment: All measurements and endpoint evaluations were conducted by a third investigator who remained blinded to group assignments and treatment protocols throughout the study. |
| Outcome measures | 6 | Parameters assessed including:  Serum biomarkers: Cardiac troponin I (cTnI), N-terminal pro-B-type natriuretic peptide (NT-proBNP);  Cardiac functional indices: LVEF, LVFS;  Histopathological evaluations: Hematoxylin-eosin (H&E) staining of cardiac tissue, and Masson's trichrome staining for cardiac fibrosis quantification;  Cardiac fibrosis-related metrics: expression of fibrosis markers of Col1, Col3, TGF-β, and α-SMA. |
| Statistical methods | 7 | Data were analyzed using GraphPad Prism 8.3 (GraphPad Software, CA, USA).  Categorical variables are presented as counts and percentages (%), while continuous variables are expressed as mean ± standard deviation (SD) or median (interquartile range, IQR).  For normally distributed numerical variables, Student’s t-test was used for comparisons between two independent groups, and one-way analysis of variance (ANOVA) was applied for multiple comparisons across three or more groups. A *P* value of less than 0.05 was deemed to indicate statistical significance. |
| Experimental animals | 8 | Species and strain: C57BL/6J mice Age/weight: 6-8 weeks old, weighing 18-22 g Sex and rationale: The exclusive use of male mice was based on two key considerations: (1) to eliminate potential confounding effects of estrogen-mediated modulation on cardiac metabolism, as demonstrated in prior mechanistic studies (PMID: 37961903), and (2) to maintain direct comparability with our established experimental models of cardiac pathophysiology (PMIDs: 37610394, 39752010, 40097836). Source and specifications: A total of 72 wild-type, specific pathogen-free (SPF) C57BL/6J mice were purchased from GemPharmatech Co., Ltd. (Jiangsu, China). |
| Experimental procedures | 9 | The therapeutic interventions were implemented following standardized protocols established in our previous investigations of ICI-associated cardiotoxicity and radiation-induced cardiac injury, and our dose exploration.  Treatment groups received either: (1) murine-derived PD-1 inhibitor (Bio X Cell, clone RMP1-14) administered intraperitoneally at 200 μg/dose (equivalent to 10 mg/kg based on average body weight) every 3 days for a total of nine doses, or (2) focal cardiac irradiation delivered as a single 16 Gy fraction using a small animal irradiator (XStrahl, Gulmay Corporation, USA). The control cohort received matched volumes of IgG2a isotype control antibody (Bio X Cell, clone 2A3) via identical administration routes paired with sham irradiation (0 Gy, identical anesthetic duration).  Reference for protocol: The selected intervention parameters were empirically derived from our prior mechanistic studies: the 16 Gy single-fraction radiation protocol was optimized to produce consistent cardiac fibrosis without acute high mortality (PMID: 37610394), while the PD-1 inhibitor dosage (200 μg/dose) was validated in our models of ICI-induced myocardial inflammation (PMIDs: 39752010, 40097836). The PD-1 inhibitor injections are administered every three days to maintain stable blood drug concentrations. All procedures were conducted during the morning circadian phase (08:00-11:00) to minimize diurnal variation effects.  Prior to the initial dose administration, comprehensive baseline assessments were performed, including: (a) transthoracic echocardiography (Vevo 2100, VisualSonics) under 1.5-2% isoflurane anesthesia to quantify left ventricular function, (b) detailed physical condition scoring (weight, fur texture, activity level). Subsequent evaluations followed our established temporal framework: acute phase (day 28, PMID: 40097836), chronic phase (3 and 5 months, PMID: 34633055) post the first dose administration. |
| Results | 10 | Statistical analyses of numerical variables between two independent groups were performed with the two-tailed Student’s t-test. For comparisons involving three or more groups, one-way analysis of variance (ANOVA) was employed. A *P* value below 0.05 was considered statistically significant. |

Note: The intervention and assessment protocols were identical for C57 female mice, IL-6 knockout (IL-6⁻/⁻) male mice, and the (Lweis lung cancer) LLC subcutaneous tumor-bearing model.

**Hepatic and renal function parameters in wild-type and IL-6 knockout mice following radioimmunotherapy.**

|  | Normal reference range | WT-iRT  (N=4) | IL-6 KO-iRT  (N=4) | *P* value |
| --- | --- | --- | --- | --- |
| Hepatic function index |  |  |  |  |
| ALT, U/L | 10.06-96.47 | 37.25 (29.74-41.46) | 25.96 (15.10-51.23) | NS, 0.7010 |
| AST, U/L | 36.31-235.48 | 161.87 (116.90-208.62) | 181.39 (157.63-36323) | NS, 0.6857 |
| D-BIL, umol/L | 0.45-33.89 | 7.39 (5.95-9.05) | 6.14 (4.21-11.87) | NS, 0.6857 |
| T-BIL, umol/L | 6.09-53.06 | 15.81 (14.20-20.27) | 14.38 (8.81-24.83) | NS, 0.8857 |
| ALB, g/L | 21.22-39.15 | 32.52 (31.21-33.34) | 31.21 (17.86-32.64) | NS, 0.4857 |
| ALP, U/L | 22.52-474.35 | 157.62 (121.73-191.79) | 123.60 (96.72-233.32) | NS, 0.6857 |
| γ-GT, U/L | 0-7.78 | 0.67 (0.50-0.77) | 0.76 (0.52-0.84) | NS, 0.3429 |
| TBA, umol/L | 0-8.51 | 2.79 (2.00-3.83) | 4.85 (4.29-7.05) | NA, 0.0571 |
| Renal function index |  |  |  |  |
| BUN, mg/dL | 10.81-34.74 | 22.53 (20.05-27.37) | 25.56 (19.98-41.08) | NS, 0.6857 |
| UREA, mmol/L | 3.9-12.4 | 8.05 (7.16-9.78) | 5.37 (2.62-8.96) | NS, 0.2000 |
| CREA, umol/L | 10.91-85.09 | 16.10 (12.85-18.05) | 21.23 (16.07-27.48) | NS, 0.1143 |
| UA, umol/L | 44.42-224.77 | 162.19 (134.05-177.14) | 202.82 (157.95-283.21) | NS, 0.2000 |

Note: Data are presented as median(Q1-Q3). Abbreviation: ALT: Alanine Aminotransferase; AST: Aspartate Aminotransferase; D-BIL:Direct Bilirubin; T-BIL: Total Bilirubin; ALB: Albumin; ALP: Alkaline Phosphatase; γ-GT: Gamma-Glutamyl Transferase; TBA: Total Bile Acids; BUN: Blood Urea Nitrogen; UREA: Urea; CREA: Creatinine; UA: Uric Acid. NS: Not significant.


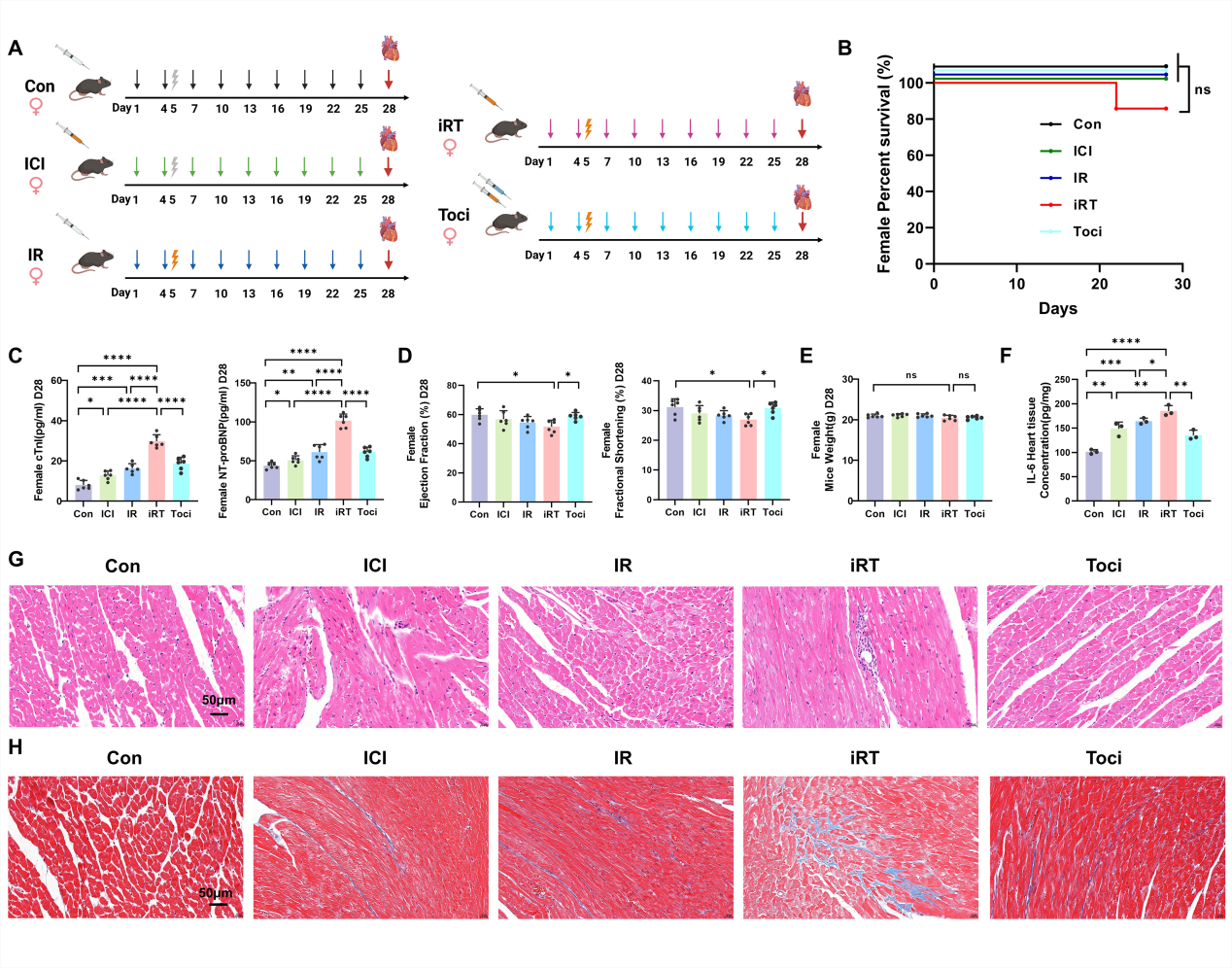


**Figure S1. CIR combined with ICI exacerbates cardiac injury in female mice.**

**A** Flowchart of the female mice model construction for cardiac injury induced by the application of radioimmunotherapy and tocilizumab treatment (n=8/group). **B** Survival curves of female mice in Con, ICI, IR, iRT and Toci groups over day 28 (n = 8/group). **C** Serum cardiac troponin I (cTnI) and N-terminal pro-B-type natriuretic peptide (NT-proBNP) levels of female mice in Con, ICI, IR, iRT and Toci groups at day 28 post-intervention (n = 6/group). **D** Left ventricular ejection fraction (LVEF) and fractional shortening (LVFS) of female mice in Con, ICI, IR, iRT and Toci groups measured by echocardiography at day 28 post-intervention (n = 6/group). **E** Changes in body weight of mice in Con, ICI, IR, iRT and Toci groups at day 28 post-intervention (n = 6/group). **F** The expression level of IL-6 of cardiac tissue of female mice in Con, ICI, IR, iRT and Toci groups at day 28 post-intervention (n = 3/group). **G** Hematoxylin and eosin (H&E) staining of cardiac tissues from female mice in Con, ICI, IR, iRT and Toci groups at day 28 post-intervention, scale bar = 50 μm. **H** Masson's trichrome staining of cardiac tissues from Con, ICI, IR, iRT and Toci groups at day 28 post-intervention, scale bar = 50 μm. ns: not significant, **P <* 0.05, ***P* < 0.01, ****P* < 0.001, *****P* < 0.0001.





**Figure S2. DEGs between groups among various types of cells in the single-cell transcriptome atlas.**

**A-F** Volcano map of DGEs of iRT vs Con, ICI vs Con and IR vs Con in fibroblasts (A), EC (B), cardiomyocytes (C), mononuclear phagocytes (MPs); (D), Bcells (E), and TandNK cell (F).


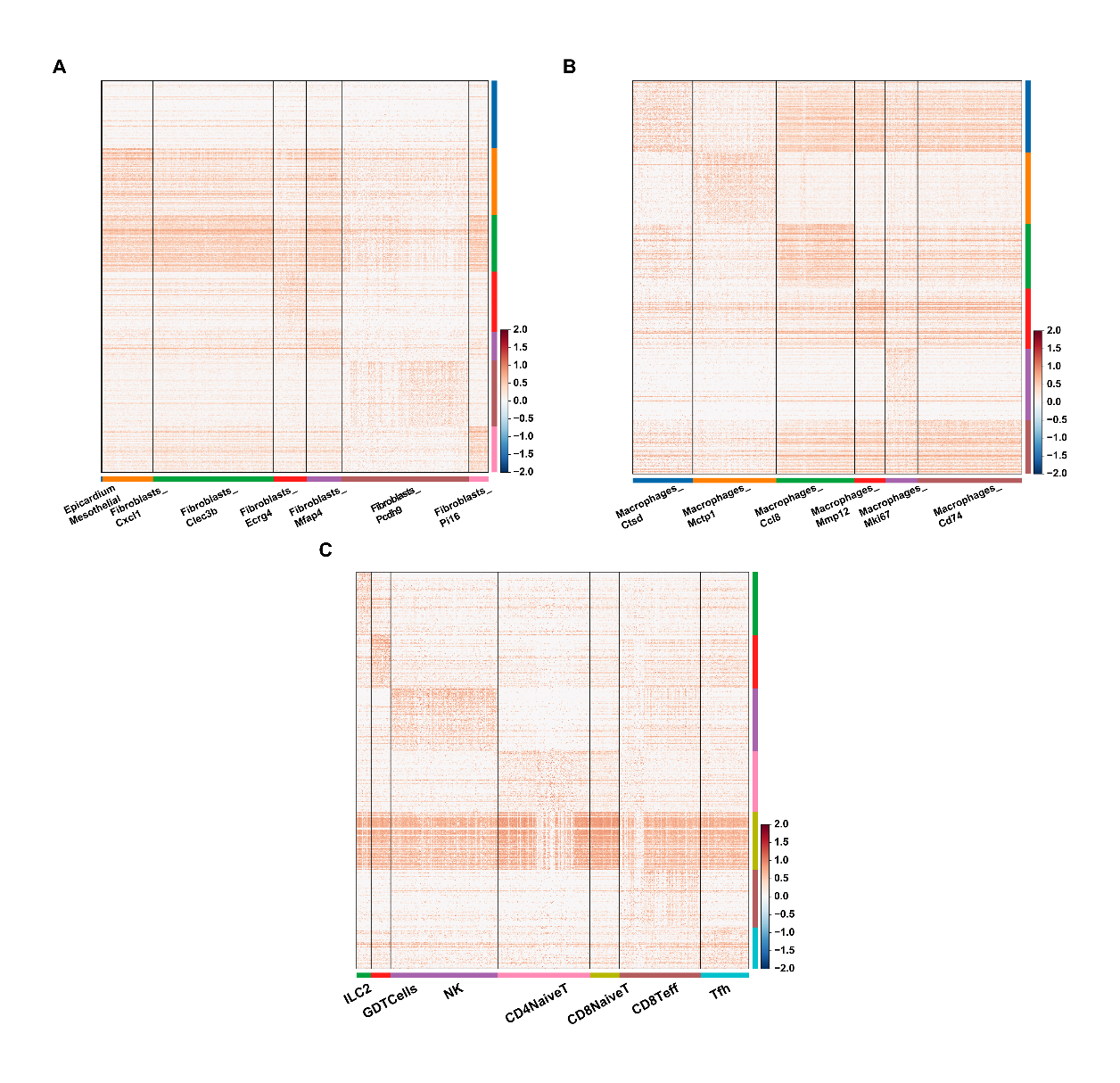


**Figure S3.** **Heatmap of DEGs among various types of cardiac cells in the scRNA-seq atlas.**

**A-C** Heatmap of the top 100 DEGs in each fibroblasts cluster (A), macrophage clusters (B), and lymphocytes clusters (C) identified through unsupervised clustering. Blue indicates lower expression, and red indicates higher expression. The expression scale is shown on the right.





**Figure S4. DEGs of fibroblast subpopulations between groups in the scRNA-seq atlas.**

**A-F** Volcano map of DGEs of iRT vs Con, ICI vs Con and IR vs Con in Clec3b fibroblasts (A), Cxcl1 fibroblasts (B), Ecrg4 fibroblasts (C), Mfap4 fibroblasts (D), Pcdh9 fibroblasts (E), and Pi16 fibroblasts subtypes (F).


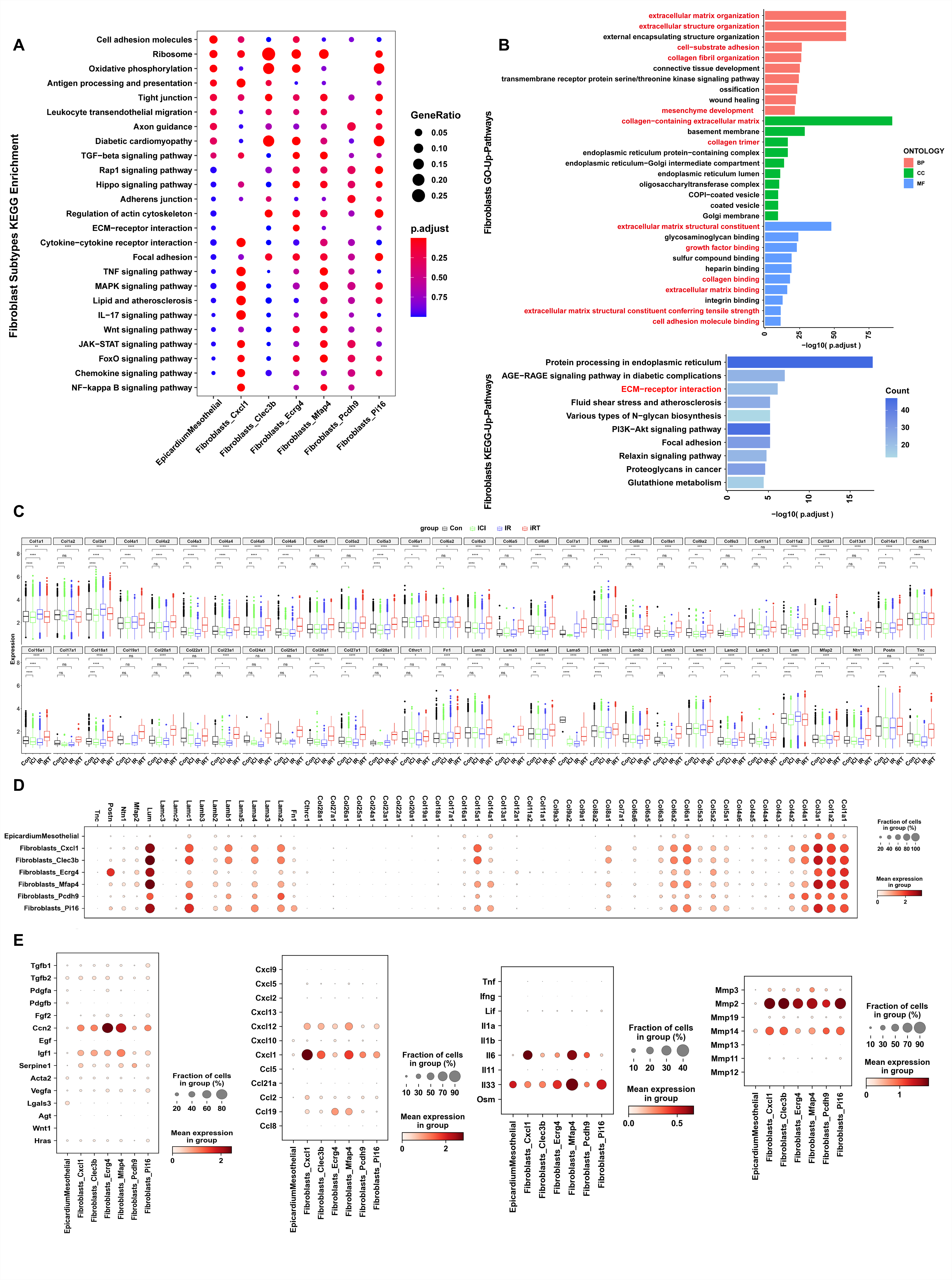


**Figure S5. Cardiac fibroblasts exhibit significant pro-fibrotic and pro-inflammatory properties in mouse hearts treated with ICI and CIR intervention.**

**A** The KEGG enrichment analysis of upregulated genes in the fibroblast subtypes. **B** GO and KEGG enrichment analysis of upregulated genes in fibroblasts. **C** The box plots showing the differential expression of ECM molecules in fibroblasts from Con, ICI, IR, and iRT groups. **D** The dot plots showing differential expression levels of ECM molecules across fibroblast subtypes in the iRT group. **E** The dot plots showing differential expression levels of pro-fibrotic and fibrinolysis-related factors across fibroblast subtypes in the iRT group. ns: not significant, **P* < 0.05, ***P* < 0.01, ****P* < 0.001, *****P* < 0.0001.


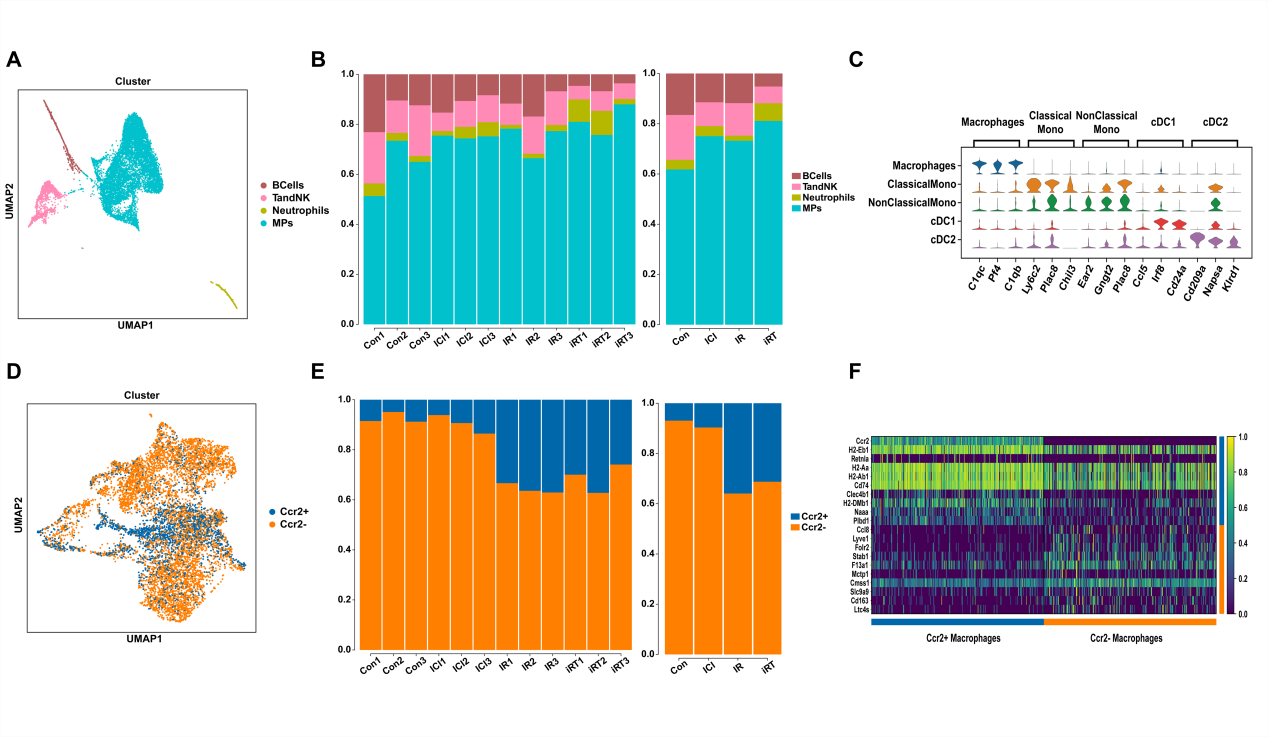


**Figure S6. The identification and characterization of subpopulations of myeloid cells.**

**A** UMAP visualization of scRNA-seq from immune cells in mouse hearts treated with ICI or/and CIR. **B** The proportion of immune subtypes in the hearts of mice across 12 mouse heart samples from Con, ICI, IR, and iRT groups (left), and after merging by intervention group (right). **C** Stacked violin plot of the top 3 marker genes for MPs subtypes. **D** UMAP visualization of scRNA-seq from CCR2^+^ and CCR2^-^ macrophages in mouse hearts treated with ICI or/and CIR. **E** The proportion of CCR2^+^ and CCR2^-^macrophages cluster in the hearts of mice across 12 mouse heart samples from Con, ICI, IR, and iRT groups (left), and after merging by intervention group (right). **F** Heatmap of the top 20 DEGs between CCR2^+^macrophages and CCR2^-^macrophages identified through unsupervised macrophages clustering. Blue indicates lower expression, and yellow indicates higher expression. The expression scale is shown on the right. CCR2: Chemokine C-C-Motif Receptor 2.





**Figure S7. DEGs of macrophages subpopulations between groups in the scRNA-seq atlas.**

**A-F** Volcano map of DGEs of iRT vs Con, ICI vs Con and IR vs Con in CCL8 macrophages (A), CD74 macrophages (B), Ctsd macrophages (C), Mctp1 macrophages (D), Mki67 macrophages (E), and Mmp12 macrophages (F).





**Figure S8. DEGs of TandNK cell subpopulations between groups in the scRNA-seq atlas.**

**A-F** Volcano map of DGEs of iRT vs Con, ICI vs Con and IR vs Con in CD4 NaiveT cell (A), CD8 NaiveT cell (B), CD8 Teff cell (C), GDT cell (D), ILC2 cell (E), and NK cell (F).

**

**

**Figure S9. Distribution and expression of immune checkpoint molecules in a mouse model of cardiac injury induced by ICI or/and CIR.**

**A** Distribution and expression of immune checkpoints molecules from cardiac cells types in Con, ICI, IR, and iRT groups visualized in the stacked violin plot. **B** Distribution and expression of Cd274, Pdcd1, Cd28, Cd86, Cd80, Ctla4, Lag3, and Pdcd1lg2 across cardiac cell types visualized in the feature plots. **C-F** Distribution and expression of Cd28 (C), Cd86 (D), Pdcd1 (E), and Cd274 (F) from cardiac cells types of the Con, ICI, IR, and iRT groups visualized in the feature plots.


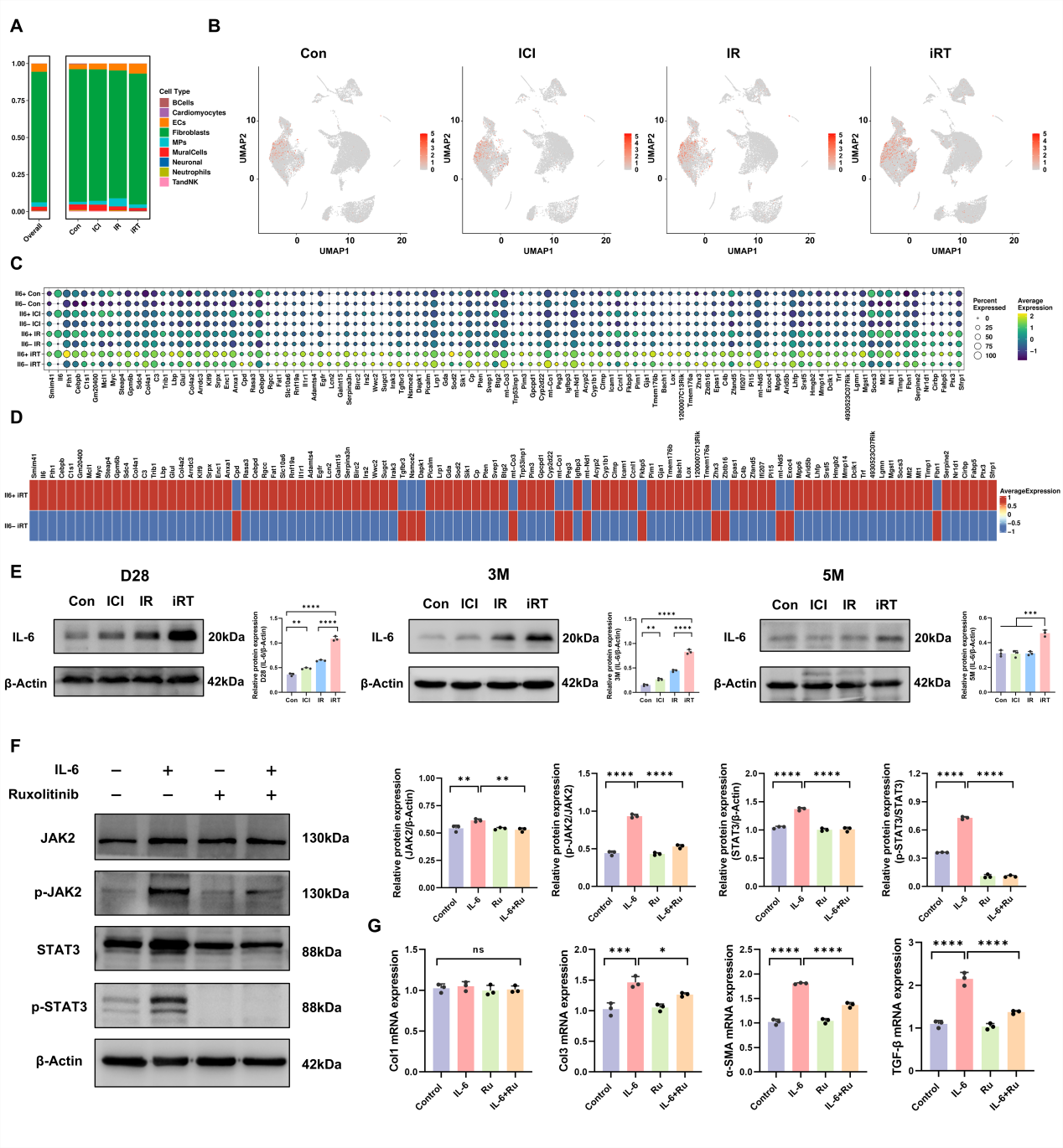


**Figure S10. Expression of IL-6 and functional effects of IL-6 in cardiac fibroblasts.**

**A** Distribution and expression of IL-6 in fibroblasts visualized in feature plot. **B** Distribution and expression of IL-6 among Con, ICI, IR, iRT groups visualized in the feature plot. **C** Heatmap showing the expression distribution of Cluster 1 genes in IL-6⁺ and IL-6⁻ fibroblasts across from the four groups. **D** Heatmap showing the expression distribution of Cluster 1 genes in IL-6⁺ and IL-6⁻ fibroblasts in the iRT group. **E** Western blot analysis of IL-6 protein levels in myocardial tissues from Con, ICI, IR, and iRT groups at day 28, 3 months, 5 months post-intervention; quantitative analysis of protein expression levels. **F** Western blot analysis of the protein levels of JAK2, phosphorylated (p)-JAK2, STAT3, and p-STAT3 in primary mouse fibroblasts stimulated by murine IL-6 cytokine with or without ruxolitinib for 48 hours; quantitative analysis of protein expression levels. **G** qRT-PCR analysis of the Col1, Col3, TGF-β, and α-SMA transcript levels in primary mouse fibroblasts stimulated by murine IL-6 cytokine with or without ruxolitinib for 48 hours. ns: not significant, **P* < 0.05, ***P* < 0.01, ****P* < 0.001, *****P* < 0.0001.


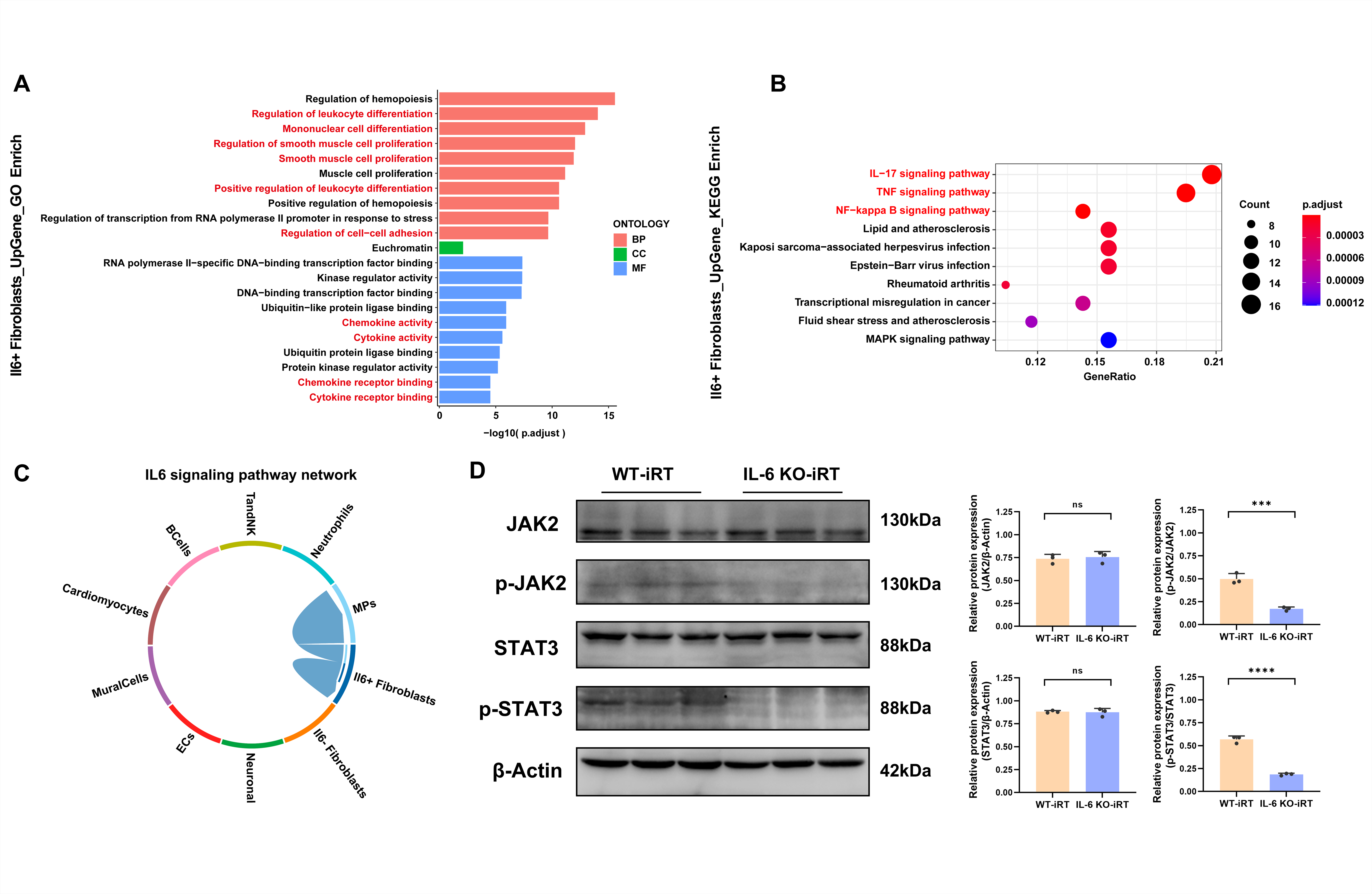


**Figure S11. Role of IL-6 positive fibroblasts in promoting cardiac inflammation and fibrosis.**

**A** GO enrichment analysis of up-regulated DEGs in IL-6^+^ fibroblasts. **B** KEGG enrichment analyses of up-regulated DEGs in IL-6^+^ fibroblasts. **C** The chord diagram depicting cell–cell interactions via the IL-6 signaling pathway between IL-6⁺ fibroblasts with other cell types in the iRT group. **D** Western blot analysis of JAK2, phosphorylated (p)-JAK2, STAT3, and p-STAT3 protein levels in hearts of wild-type (WT) and IL-6–knockout (KO) mice treated with radioimmunotherapy; quantitative analysis of protein expression levels (n = 3/group). ns: not significant, ****P* < 0.001, *****P* < 0.0001.


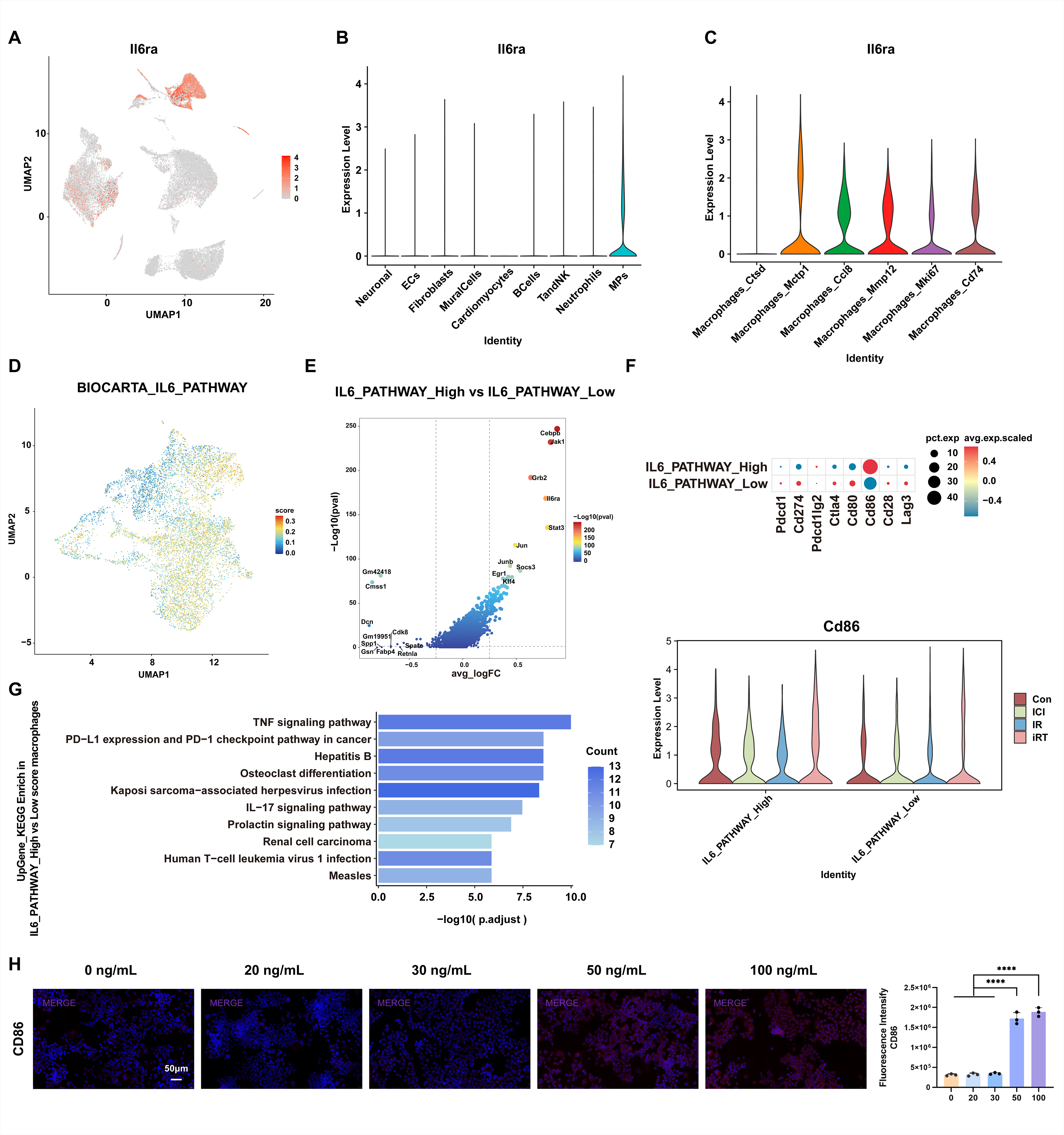


**Figure S12. Expression of IL-6RA and functional effects of IL-6 signaling in cardiac macrophages.**

**A–C** Distribution and expression of IL-6RA across nine major cardiac cell types (A, B) and macrophage subtypes (C). **D** UMAP visualization of macrophages colored according to IL-6 signaling pathway activity score. **E** Volcano plot of DEGs between macrophages with high vs low IL-6 signaling activity. **F** Expression levels of immune checkpoint molecules in macrophages with high vs low IL-6 pathway activity. **G** KEGG enrichment analysis of up-regulated DEGs in macrophages with high IL-6 signaling activity. **H** CD86 surface expression levels in macrophages incubated with different concentrations of murine IL-6, scale bar = 50 μm. ns: not significant, **P* < 0.05, ***P* < 0.01, ****P* < 0.001, *****P* < 0.0001.


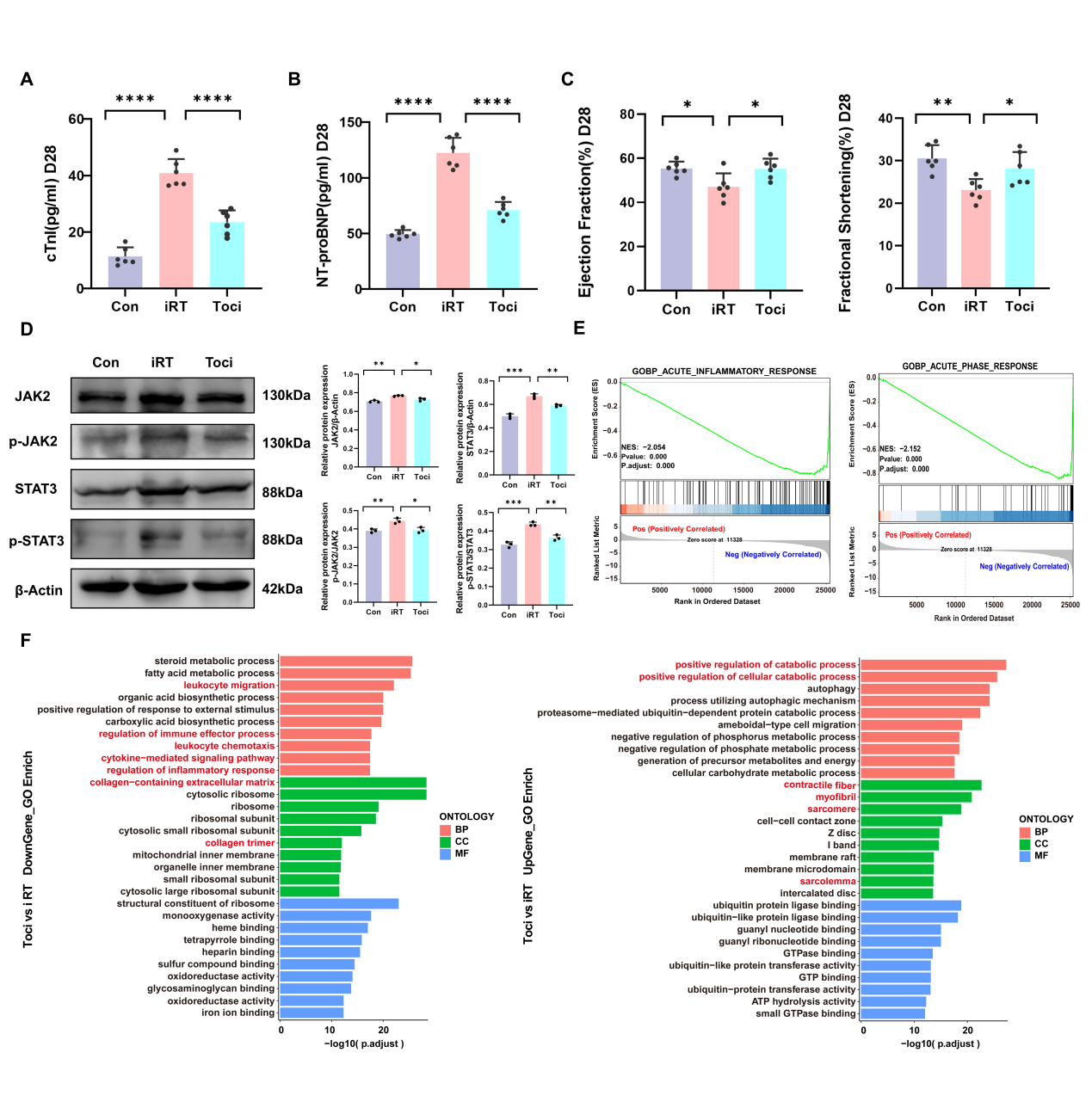


**Figure S13. Tocilizumab attenuates radioimmunotherapy-induced cardiac injury and fibrosis.**

**A** Serum cTnI levels measured by ELISA in mice from Con, iRT and Toci groups at day 28 (n = 6/group). **B** Serum NT-proBNP levels measured by ELISA in mice from Con, iRT and Toci groups at day 28 (n = 6/group). **C** Echocardiographic assessment of left ventricular function at day 28, including LVEF and LVFS in mice from Con, iRT, and Toci groups (n = 6/group). **D** Western blot analysis of JAK2, phosphorylated (p)-JAK2, STAT3, and p-STAT3 protein levels in cardiac tissues from Con, iRT, and Toci groups; quantitative analysis of protein expression levels (n = 3/group). **E** Gene Set Enrichment Analysis (GSEA) functional enrichment analysis in Toci vs iRT group. **F** GO functional enrichment analysis of up-regulated (right) and down-regulated (left) DEGs in Toci vs iRT group. ns: not significant, **P* < 0.05, ***P* < 0.01, ****P* < 0.001, *****P* < 0.0001.


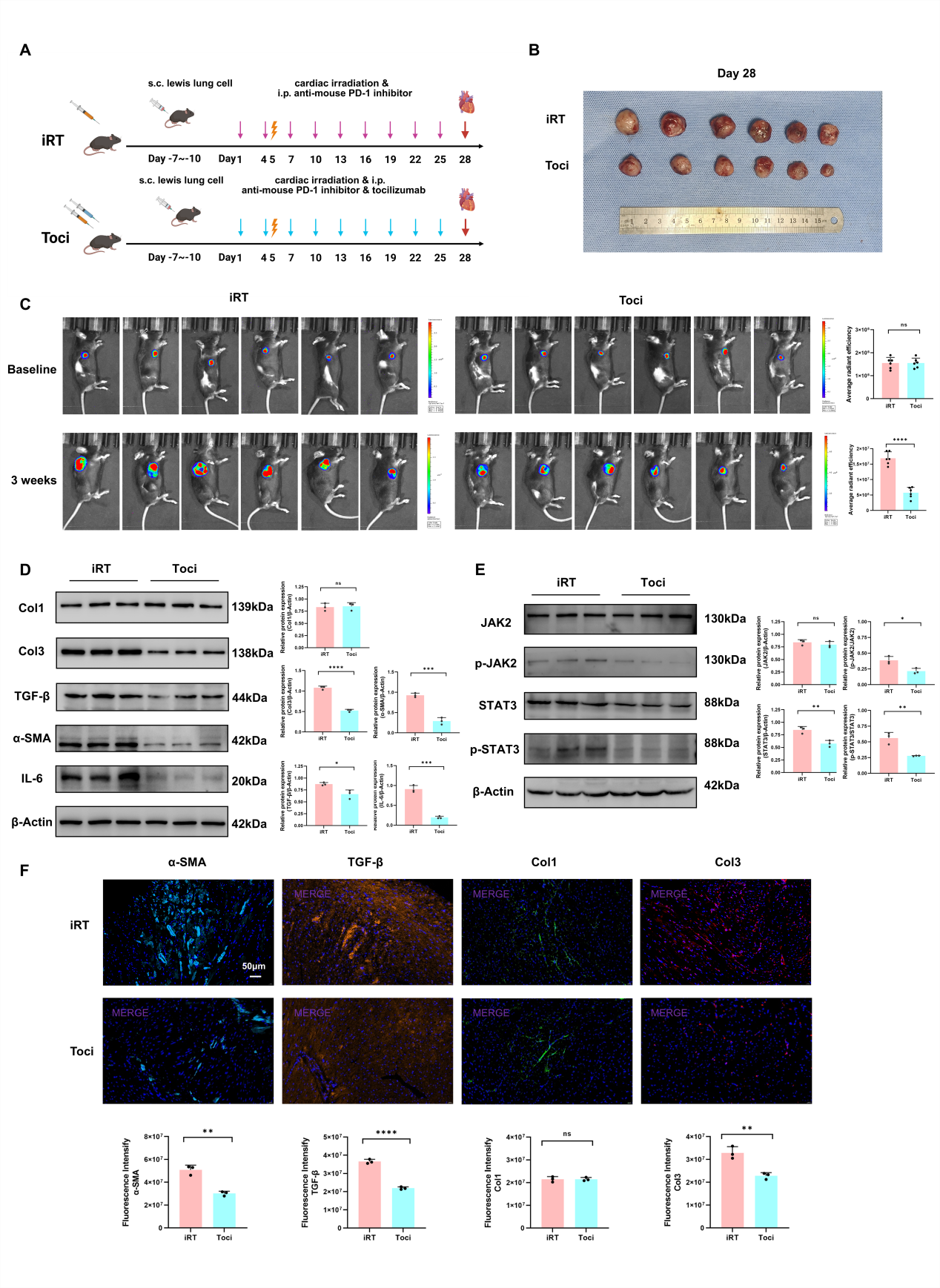


**Figure S14. The effect of tocilizumab on cardiac fibrosis and antitumor efficacy in a lewis lung cancer (LLC)-bearing mouse model** **induced by radioimmunotherapy.**

**A** Schematic of the IL-6 RA inhibitor tocilizumab intervention in radioimmunotherapy-induced cardiac injury mouse model bearing LLC cells. **B** Images of mouse tumors in iRT and Toci groups at day 28 post-intervention. **C** Bioluminescence imaging was performed on mice from iRT and Toci groups at baseline and 3 weeks post-intervention (n = 6 /group); In vivo imaging tumor measurement of average radiant efficiency. **D** Western blot analysis of Col1, Col3, α-SMA, TGF-β, and IL-6 protein levels in myocardial tissues from iRT and Toci groups at 28 days post-intervention; quantitative analysis of protein expression levels (n = 3/group). **E** Western blot analysis of JAK2, phosphorylated (p)-JAK2, STAT3, and p-STAT3 protein levels in cardiac tissues from iRT and Toci groups at 28 days post-intervention; quantitative analysis of protein expression levels (n = 3/group). **F** IF staining of Col1, Col3, α-SMA, TGF-β, and IL-6 in cardiac tissues of Con, iRT, and Toci groups at 28 days post-intervention; quantitative histograms of corresponding markers, scale bar = 50 μm. ns: not significant, **P* < 0.05, ***P* < 0.01, ****P* < 0.001, *****P* < 0.0001.


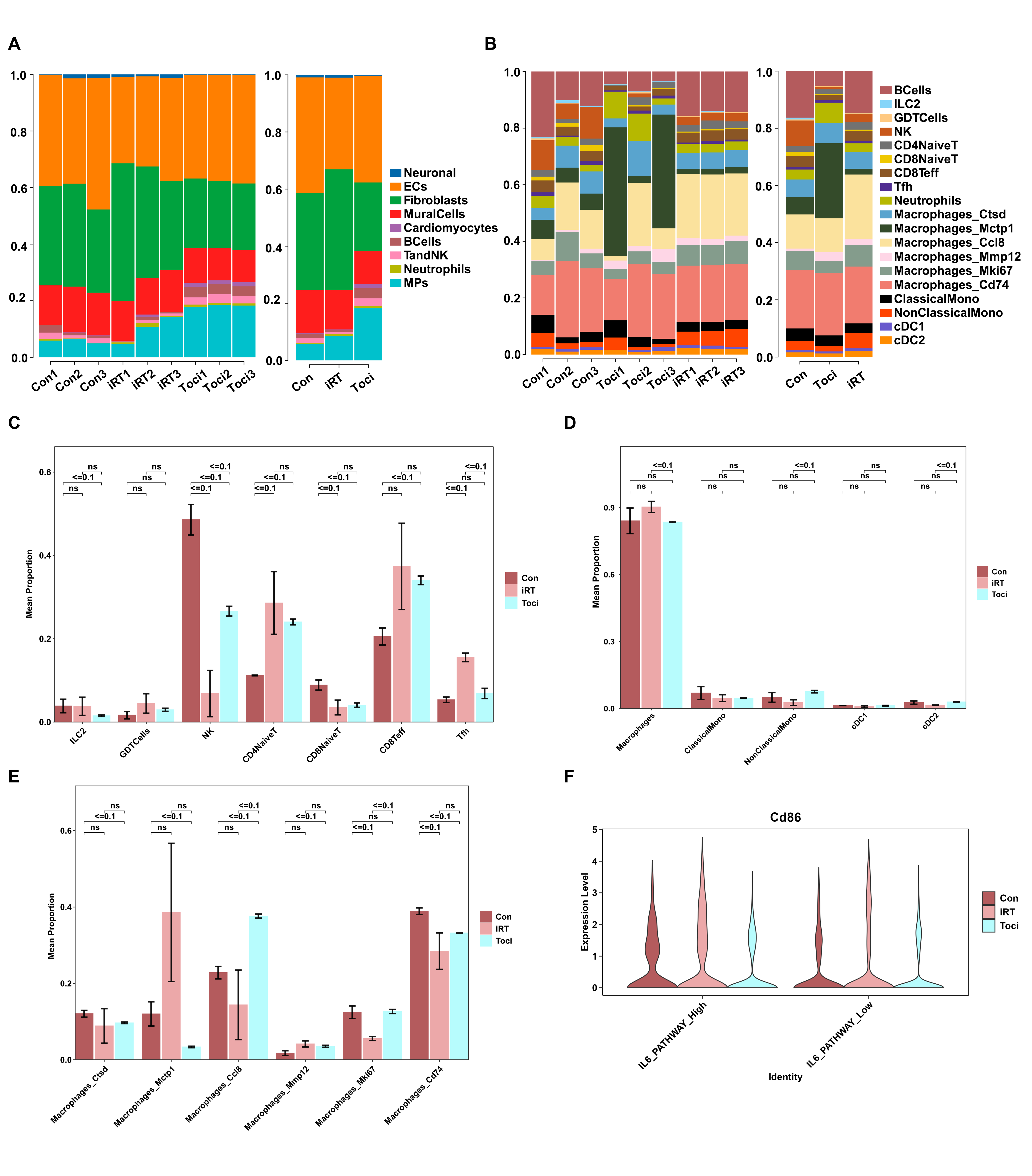


**Figure S15. Cellular composition and proportions in mouse hearts after tocilizumab treatment in combination with radioimmunotherapy.**

**A** The proportion of nine cell types in the hearts of mice across 9 mouse heart samples from Con, iRT, and Toci groups (left), and after merging by intervention group (right). **B** The proportion of immune cell subtypes in mice hearts across 9 mouse heart samples from Con, iRT, and Toci groups (left), and after merging by intervention group (right). **C** Bar plots showing the proportional distribution of TandNK cell subtypes in Con, iRT, and Toci groups. **D** Bar plots showing the proportional distribution of MPs subtypes in Con, iRT, and Toci groups. **E** Bar plots showing the proportional distribution of macrophage subtypes in Con, iRT, and Toci groups. **F** Expression levels of immune checkpoint molecule of CD86 in macrophages with high vs. low IL-6 pathway activity across the Con, iRT, and Toci groups. ns: not significant, **P* < 0.05, ***P* < 0.01, ****P* < 0.001, *****P* < 0.0001, n=3/group.
